# Phylogenetic relationships of *Atractylodes lancea*, *A*. *chinensis* and *A*. *macrocephala*, revealed by complete plastome and nuclear gene sequences

**DOI:** 10.1371/journal.pone.0227610

**Published:** 2020-01-28

**Authors:** Liqiang Wang, Hui Zhang, Xi Wu, Ziyue Wang, Weiwei Fang, Mei Jiang, Haimei Chen, Linfang Huang, Chang Liu

**Affiliations:** 1 Key Laboratory of Bioactive Substances and Resource Utilization of Chinese Herbal Medicine, Ministry of Education, Institute of Medicinal Plant Development, Chinese Academy of Medical Sciences and Peking Union Medical College, Haidian, Beijing, China; 2 School of Computer and Information Technology, Beijing Jiaotong University, Beijing, China; National Cheng Kung University, TAIWAN

## Abstract

*Atractylodes lancea*, *A*. *chinensis*, and *A*. *macrocephala* are the three most widely used medicinal species of the *Atractylodes* genus. Their similar morphological features cause disagreement as whether they are three unique species, leading to their frequent misuses in medical products. Our study aimed to understand their relationships through both the complete plastome sequences and nuclear sequences, to identify molecular markers for their differentiation and explore the evolutionary relationships among three species. We sequenced, annotated, and analyzed the plastomes of these three species. The plastomes are 153,201, 153,258, and 153,265 bps in length for *A*. *lancea*, *A*. *chinensis*, and *A*. *macrocephaly*, respectively. Similar to other Asteraceae species, their plastomes exhibit typical quadripartite structures. Each plastome consists of 119 distinct genes, including 78 protein-coding, 37 tRNA, and 4 rRNA genes. Analyses of indels, single-nucleotide polymorphisms and simple sequence repeats, and comparison of plastomes showed high degree of conservation, leading to difficulty in the discovery of differentiating markers. We identified eleven potential molecular markers using an algorithm based on interspecific and intraspecific nucleotide diversity gaps. Validation experiments with fifty-five individuals from the three species collected from the botanical garden and fields confirmed that the marker cz11 could effectively distinguish samples from the three different species. Analysis of the several nuclear sequences suggests that the species of *A*. *macrocephala* may be a hybrid *of A*. *lancea* and *A*. *chinensis*. In summary, the results from this study highlight the complex relationships among of these three medicinal plants.

## Introduction

The genus *Atractylodes* (family Asteraceae) consists of seven species which are distributed across China, Korea, and Japan. Several *Atractylodes* species have long been known for their medicinal values. According to clinical purposes, their dried rhizomes are used as two kinds of herbal medicines, namely, Baizhu in Chinese or byakujutsu in Japanese and Cangzhu in Chinese or sojutsu in Japanese [1, 2]. Among these speices, only *A*. *carlinoides* (Hand.-Mazz.) Kitam. has a stoloniferous and slender rhizome. Many other *Atractylodes* species have thick rhizomes. In particular, the thick rhizomes of *A*. *lancea* (Thunb.) DC. and *A*. *chinensis* (Bunge) Koidz. have been classified as Cangzhu and that of *A*. *macrocephala* Koidz. has been classified as Baizhu in the pharmacopeias of China, Korea, and Japan [3]. In traditional Chinese medicine, the pharmacological effects of Baizhu and Cangzhu are distinct. Baizhu causes diaphoretic activities, and Cangzhu causes antisudorific activities. Recent studies have shown that rhizomes of *Atractylodes* exhibit many other pharmacological effects, such as antibacterial, immunomodulatory, antitumor, and antiosteoporosis, thereby indicating the considerable potential of *Atractylodes* in the development of new drugs [4, 5].

Despite the wide usage of species of *Atractylodes* as source of medicinal products, controversies exist over the taxonomic relationships among its species [1, 3]. Furthermore, different species of *Atractylodes* possess different chemical constituents with diverse bioactivities. Accurate species identification is essential to ensure the clinical safety of medicinal products derived from *Atractylodes* plants. However, given the morphological similarity, distinguishing the species by macroscopic observation is difficult. Consequently, the misuse of rhizomes of *Atractylodes* is common when species identification is based only on morphological features. Therefore, an accurate method of species determination is required to ensure the safety and effective uses of *Atractylodes* species for medicinal purposes.

Many molecular identification methods have been developed in the past years [6]. Recently, the universal DNA barcodes, such as internal transcribed spacer (ITS) regions and *trn*K gene [7], have been used for species identification and phylogenetic analysis of *Atractylodes* species [1, 8]. Nevertheless, these markers have not performed satisfactorily at the species and intraspecific levels due to unclear species boundaries and low resolution [1, 3]. Hence, additional markers should be developed. A complete chloroplast genome is a rich source of additional molecular markers. The chloroplast genomes are more conserved than the nuclear genomes in plants. Nonetheless, many mutational events, including insertions/deletions (indels), substitutions, and inversions in the chloroplast DNA sequence, have been identified [9, 10]. These mutations can be used as markers to rapidly distinguish species [11]. Furthermore, these mutations can resolve complex evolutionary relationships and improve the resolution at low taxonomic levels among closely related species [12–15].

With the rapid advancement in next-generation sequencing (NGS) technologies, DNA sequencing becomes affordable in regular laboratories. Along with genome-skimming strategies, obtaining complete chloroplast DNA (cpDNA) sequences at low cost without prior purification of cpDNA is easy [16]. The present study aims to determine the phylogenetic relationship of several *Atractylodes* species and identify molecular markers for their differentiation.

Chloroplasts are a kind of plastid and are present in multiple copies in a cell. In the following text, we will refer chloroplast genome as plastome. In our initial analysis, we found that the plastome regions that can be used to distinguish among closely related species commonly display large intraspecific variations, but they can yield low success rates in DNA sequencing. To identify the DNA barcodes for the differentiation of closely related species, we used a computational algorithm, called the *Sequence Diversity Gap Analyzer* (SeqDivGap), which can rank all regions based on the scale of an index called Diversity Gap (data not shown). Particularly, the Diversity Gap was defined as the difference between inter- and intra-specific diversity of a DNA region. It is conceptually similar to DNA barcoding gap, but with different calculation methods [17].

Because plastids are maternally inherited in most plant species, nuclear genomes have several advantages in identifying the real relationship among species, especially if there were hybridization among different species. However, it is still rather expansive to obtained the complete nuclear genome sequence of a species, as a result, target enrichment has been used to generate a subset of all genes for phylogenetic comparison [14]. In particular, a set of probes to capture 353 low-copy nuclear genes have been designed to work across angiosperms for classification and identification [18].

In summary, a combined genome skimming strategy was used to obtain the complete plastomes of three *Atractylodes* species. The plastomes were characterized in detail and we identified a molecular marker that can be used to distinguish the three closely related species based on the ranking of the index Diversity Gaps. Comparison of the nuclear genes identified one sequence suggesting that *A*. *macrocephala* may be a hybrid species of *A*. *lancea* and *A*. *chinensis*. The plastome DNA markers, and nuclear gene sequences reported here could provide a valuable genetic resource for genetic diversity, phylogenetic evolution and taxonomy studies of the Asteraceae family.

## Materials and methods

### Plant materials and total DNA extraction and sequencing

We collected fresh leaves of *A*. *lancea*, *A*. *chinensis*, and *A*. *macrocephala* from the Institute of Medicinal Plant Development (IMPLAD), Beijing, China. All samples were identified by Professor Zhao Zhang of IMPLAD. The voucher specimens were deposited in the herbarium of the IMPLAD (S1 Table). Total DNA was extracted using a plant genomic DNA extraction kit (Tiangen Biotech, Beijing). DNA quality was assessed by electrophoresis in 1% (w/v) agarose gel, and the quantity was examined using Qubit 3.0 (Life Technologies, Carlsbad, CA, USA). Approximately 500 ng of DNA was used to construct a library with insert size of 500 bps, and it was sequenced according to the manufacturer’s instructions for MiSeq platform (Illumina Inc., San Diego, CA). A total of 5.8, 5.9, and 6.0 Gbs of raw data from *A*. *lancea*, *A*. *chinensis*, and *A*. *macrocephala*, respectively, were produced with 250 bps pair-end read lengths. The raw data was deposited in the Sequence Read Archive (SRA) under the BioProject accession number of PRJNA556560.

### Plastome assembly and annotation

The paired-end reads were filtered against all plastome sequences available in the National Center for Biotechnology Information (NCBI) using BLASTN with an e-value cutoff of 1e-5. The extracted reads were assembled using SPAdes (v3.10.1), and the resulting contigs were extended by a python script [19]. Afterward, the extended contigs were further assembled by the Seqman module of DNAStar (v6.10.01) [20]. The correctness of the complete draft plastome was validated by mapping all raw reads against the reference genomes using Bowtie 2 (v2.0.1) [21]. Results were visualized using Tablet [22]. Gene annotation was performed using CpGAVAS2 web service [23–25], and the initial annotations were edited manually by Apollo genome editor [26]. The circular map was generated using OrganellarGenomeDRAW [27].

### Genome feature identification and comparative analysis

The REPuter web service was used to identify four types of sequence repeats, including forward, palindromic, reverse, and complement repeats [28]. The minimal repeat size was set at 30 bp, and the cutoff for similarities among the repeat units was set at 90%. Simple sequence repeats (SSRs) were predicted using MISA Perl Script with the following thresholds: eight units of mononucleotides; four units of di- and tri-nucleotides; and three units of tetra-, penta-, and hexa-nucleotides [29]. Indels and single nucleotide polymorphisms (SNPs) were analyzed on the basis of sequence alignments using DnaSP version 5.1 [30]. We compared the plastomes of three *Atractylodes* species by using the software mVISTA in Shuffle-LAGAN mode with the plastome of *A*. *lancea* as the reference [31]. The codon usage distribution was investigated by using the software CodonW (University of Texas, Houston, TX, USA).

### Phylogenetic analyses

To determine the phylogenetic position of the three *Atractylodes* species, we downloaded 37 complete plastome protein-coding sequences from GenBank, including those of *Nymphoides coronata* and *Menyanthes trifoliata* from Menyanthaceae as outgroups (S2 Table). A total of 64 common protein-coding gene sequences in all of the 40 species were obtained by manual detection (PSBA, MATK, RPS16, PSBK, PSBI, PETN, PSBM, RPOB, RPOC2, RPS2, ATPI, ATPH, ATPF, ATPA, PSBD, PSAB, PSAA, YCF3, RPS4, NDHJ, NDHK, NDHC, ATPE, ATPB, PSAI, YCF4, CEMA, PETA, PSBJ, PSBF, PSBE, PETL, PETG, PSAJ, RPL33, RPS18, RPL20, RPS12, CLPP, PSBT, PSBN, PSBH, PETB, PETD, RPS11, RPL36, RPS8, RPL14, RPL16, RPS3, RPL22, RPL23, RPS7, RPS15, NDHH, NDHA, NDHI, NDHG, NDHE, PSAC, NDHD, CCSA, RPL32, NDHF). We aligned these sequences using the ClustalW algorithm [32]. The phylogenetic tree was constructed using maximum likelihood method implemented in the software RAxML [33]. The parameters were “raxmlHPC-PTHREADS-SSE3 -f a -N 1000 -m PROTGAMMACPREV/GTRGAMMA -x 551314260 -p 551314260 -o NC_041436,NC_041484 -T 20”. The significant level of the phylogenetic tree was assessed by bootstrap testing with 1000 replications.

### Identification and validation of molecular markers for the three *Atractylodes* species

To authenticate the three *Atractylodes* species, we developed an algorithm called seqDivGap (not published) for the research of molecular markers. On the basis of the results predicted by the seqDivGap software, we selected seven regions with the highest likelihood of containing good molecular markers. Eleven pairs of primers were designed using NCBI’s Primer BLAST tool (S3 Table). We collected 5 individuals from each species from the botanical garden in IMPLAD. DNA samples were extracted and then subjected to PCR amplification using the seven pairs of primers on a Pro Flex PCR system (Applied Biosystems, Waltham, MA, USA). The PCR experiments were conducted under the following conditions: pre-denaturation at 94 °C for 2 min, 35 cycles of amplification at 94 °C for 30 s, 55 °C for 30 s, and 72 °C for 30 s, followed with a final extension at 72 °C for 2 min. The PCR reaction mixture contained 12.5 μl of Taq MasterMix (2 ×), 1 μl of forward primer (10 μM), 1 μl of reverse primer (10 μM), and purified chloroplast DNA (< 1 μg) [8]. The PCR products were evaluated with 1% agarose gel electrophoresis. Only single bands were subjected to Sanger sequencing. In addtion, we collected 40, 18 and 18 samples from the markers that claimed to be *A*. *lancea*, *A*. *chinensis* and *A*. *macrocephala* respectively. These samples were analyzed following the same procedure described above except that only the one primer pair cz11 were used.

### Variation analysis for target sequences

We mapped all the reads to the target (marker) sequence to determine the diversity levels at a particular locus. Mapping was conducted by extracting a fragment from the plastome with a total length of 600 bps and the target region in the middle. This sequence was used to search against the NGS reads produced previously for the three species using BLASTN with an e-value cutoff of 1e-5. The reads passing the cutoff value were selected and aligned to the target sequence by using Clustalw2. Finally, the alignments were extracted using the extractalign program in the EMBOSS package [34]. The types and frequencies of reads mapped to the target sequence for each species were calculated.

### Analysis of the relationship of three *Atractylodes* species with nuclear genes

A universal probe set of 353 nuclear genes from any flowering plant designed from Matthew GJ et al [18] were used as our target sequences. The length of the probes was 80 to 120 bps, which came from 42 angiosperms and have been tested useful in 283 species. The probe sequences are publicly available under a CC-BY-SA license at github.com/mossmatters/Angiosperms.

We used the pipeline HybPiper (v1.2) (https://github.com/mossmatters/HybPiper) with the default settings to process our cleaned sequence data [14]. Briefly, reads were mapped to target probe sequences using BWA. And those reads that were successfully mapped were assembled into contigs using SPAdes. The assembled contigs are aligned to the target *Atractylodes* gene sequence using Exonerate. Ideally, HybPiper identifies a single contig corresponding to each *Atractylodes* gene sequence. However, if paralogs exist, SPAdes might produce multiple contigs, each corresponding to one paralog of the target *Atractylodes* gene. Phylogenetic tree was constructed with nuclear genes by using RaxML with 1000 bootstrap replicates.

## Results

### Structural organization of the three plastomes

**Fig 1:**
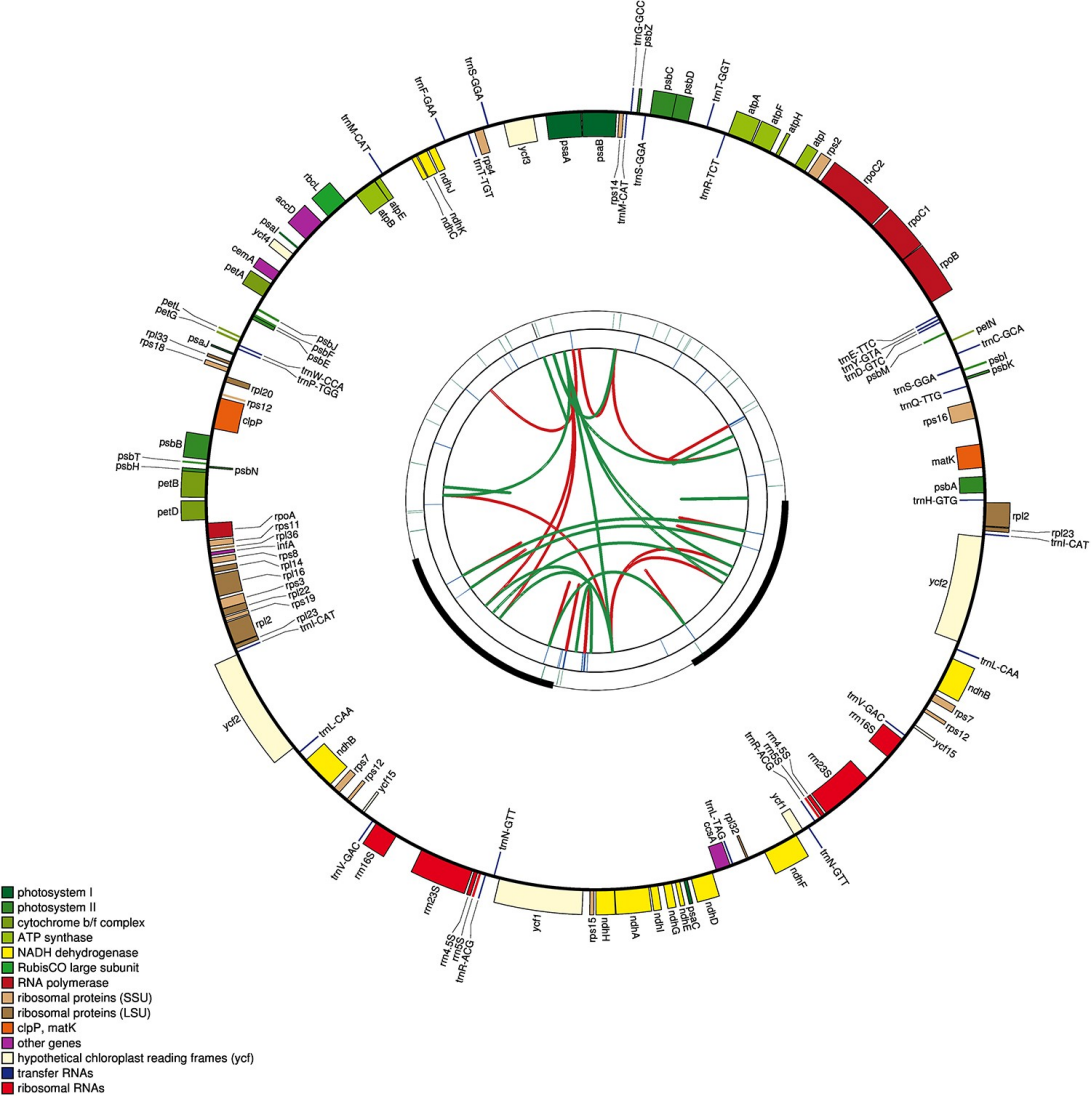
Graphic representation of features identified in the plastome of three *Atractylodes* species by using CPGAVAS2. The map contains four rings. From the center going outward, the first circle shows the forward and reverse repeats connected with red and green arcs, respectively. The next circle shows the tandem repeats marked with short bars. The third circle shows the microsatellite sequences identified using MISA. The fourth circle is drawn using drawgenemap and shows the gene structure on the plastome. The genes were colored based on their functional categories, which are shown at the left corner.

We obtained the plastomes of *A*. *lancea*, *A*. *chinensis*, *and A*. *macrocephala* using the genome-skimming strategy. The sequences have been deposited in GenBank (accession numbers: MG874804, MG874805, and MN661162. The schematic representations of the three plastomes are shown in Fig 1, and their general features are presented in Table 1. *A*. *lancea*, *A*. *chinensis*, and *A*. *macrocephala* show total lengths of 153,201, 153,258, and 153,261 bps, respectively. All three genomes display the typical quadripartite structure with pairs of inverted repeats (IRs) of 25,148, 25,148 and 25,154 bps in length separated by large single-copy (LSC) regions of 84,249, 84,282 and 84,280 bps long and small single-copy (SSC) regions of 18,656, 18,680 and 18,673 bps in length, respectively.

**Table 1:** Summary of the plastome features for the three *Atractylodes* species.

	Feature	*A*. *chinensis*	*A*. *macrocephala*	*A*. *lancea*
**Length (bp)**	Total	153258	153261	153201
	LSC	84282	84280	84249
	SSC	18680	18673	18656
	IR	25148	25154	25148
**GC content (%)**	Total	37.7	37.7	37.7
	LSC	35.8	35.8	35.8
	SSC	31.5	31.5	31.5
	IR	43.2	43.2	43.2
**No. of genes**	Total	132	130	132
	Protein coding	87	85	87
	tRNA	37	37	37
	rRNA	8	8	8

IR = Inverted repeat region; LSC = Large single-copy region; SSC = Small single-copy region.

Overall, the GC content is 37.7% in all three species. This value is lower than that of IR regions (43.2%) and higher than that of the LSC (35.8%) and SSC regions (31.5%), which suggested that the LSC, SSC, and IR regions may present different origins or selection pressures. The high GC content in the IR regions is attributed to the four rRNA genes with high GC content in the regions.

With regard to gene content, the three genomes are highly conserved. *A*. *lancea and A*. *chinensis* contain 132 genes, *while A*. *macrocephala* has 130 genes, all of them include 119 distinct genes, and encode 78 proteins, 37 tRNAs, and four rRNAs (Table 2). Seven genes, namely, *rpl*23, *ycf*1, *ycf*2, *ycf*15, *ndh*B, *rps*7, and *rps*12, and all rRNA genes are found in the IR regions (Fig 1). The genome shows 21 intron-containing genes, including 11 protein-coding genes and 8 tRNA genes with one intron and two protein-coding genes (*clp*P and *ycf*3) with two introns (S4 Table) in all three species. The *rps*12 gene is a special trans-splicing gene: the 5' exon is in the LSC region, and the 3' exon is located in the IR region. This arrangement is similar to those found in other plant species, such as *Olea europaea* L. [14].

**Table 2:** Gene contents of the three Atractylodes plastomes.

No.	Group of genes	Gene names	Amount
**1**	ATP synthase	*atp*A, *atp*B, *atp*E, *atp*F, *atp*H, *atp*I	6
**2**	Photosystem II	*psb*A, *psb*B, *psb*C, *psb*D, *psb*E, *psb*F, *psb*I, *psb*J, *psb*K, *psb*M, *psb*N, *psb*T, *psb*Z, *ycf*3	14
**3**	NADH-dehydrogenase	*ndh*A, *ndhB*(×2), *ndh*C, *ndh*D, *ndh*E, *ndh*F, *ndh*G, *ndh*H, *ndh*I, *ndh*J, *ndh*K	12
**4**	Cytochrome b/f complex	*pet*A, *pet*B, *pet*D, *pet*G, *pet*L, *pet*N	6
**5**	Photosystem I	*psa*A, *psa*B, *psa*C, *psa*I, *psa*J	5
**6**	Rubisco large subunit	*rbc*L	1
**7**	Transfer RNAs	37 tRNAs (eight contains one intron, seven in the IRs)	37
**8**	Ribosomal RNAs	*rrn*16S(×2), *rrn*23S(×2), *rrn*4.5S(×2), *rrn*5S(×2)	8
**9**	RNA polymerase	*rpo*A, *rpo*B, *rpo*C1, *rpo*C2,	4
**10**	Small ribosome subunit	*rps*2, *rps*3, *rps*4, *rps*7(×2), *rps*8, *rps*11, *rps*12(×2), *rps*14, *rps*15, *rps*16, *rps*18, *rps*19	14
**11**	Large ribosome subunit	*rpl*14, *rpl*16, *rpl*2(×2), *rpl*20, r*pl*22, *rpl*23(×2), *rpl*32, *rpl*33, *rpl*36	11
**12**	Other genes	*acc*D, *ccs*A, c*em*A, *clp*P, *inf*A, *mat*K	6
**13**	Proteins of unknown function	*ycf*1 (×3*, ×1**), *ycf*2(×2), *ycf*4, *ycf*15(×2)	8*/6**

*: *A lancea and A*. *chinensis*;

**: *A*. *macrocephala*.

A total of 87 protein-coding genes coding for 10,922 codons in the plastomes are observed in the three plant species. Among these codons, isoleucine and cysteine show the highest and lowest frequencies represented by 1,152 (10.55%) and 114 (1.04%) codons, respectively (S5 Table). The codon usage of protein-coding genes in the three plastomes is summarized in S5 Table. The relative synonymous codon usage (RSCU) values of the same codon among the three species are similar (S1 Fig). In addition, the A/T contents for the first, second, and third codon positions within the protein-coding regions (CDS) of the plastomes are 58.32%, 63.18%, and 68.20%, respectively. Evidently, a bias of high A/T ratio at the third codon position is detected, which is similar to those observed in other land plants [18].

### Repeat analyses in the three plastomes

Repeat sequences play an important role in the creation of genetic diversity in terms of genome lengths and structural rearrangement [35]. We analyzed the structure and distribution of the repeats in the plastomes of *A*. *lancea*, *A*. *chinensis*, *and A*. *macrocephala*, and the details are shown in S6 Table. Correspondingly, a total of 39, 37, and 39 repeats longer than 30 bps were detected in the three plastomes, respectively, with similarities higher than 90%. Results revealed similarities in the lengths and number of repeats across the three plastomes. *A*. *lancea* shows 19 forward and 20 palindromic repeats, *A*. *chinensis* displays 18 forward and 19 palindromic repeats, and *A*. *macrocephala* exhibits 19 forward and 20 palindromic repeats. Most repeats are distributed within the IGS, and a majority of repeats show lengths between 30 bps and 40 bps (S2 Fig).

SSRs are tandemly repeated DNA sequences consisting of 1–6 nucleotide repeat units, also known as microsatellites, which are distributed throughout the plastomes [36]. SSRs are widely used as molecular markers in population genetics, species identification, and phylogenetic investigations based on their high-degree variations [37]. We identified a total of 48 SSRs in the three plastomes after the analyses (S7 Table). The SSRs are mostly distributed in the IGS and intron sequences. Most mononucleotide repeats consist of A/T repeats, and the AT/AT dinucleotide repeats are the most common type. These results are in accordance with the previous report that SSRs from plastomes are generally composed of short polyA or polyT repeats, and they rarely contain tandem G or C repeats in many plants [38]. In total, nine SSRs were identified in the CDS of six genes in the three species, including *rpo*B, *rpo*C1, *rpo*C2, *psb*C, *rpo*A, and *ycf*1. Results indicated that the three plastomes are highly conserved in terms of the numbers and the GC content of SSRs.

### Discovery of indels and variation sites among the three plastomes

Indels and SNP sites are common events in the evolution of higher-plant plastomes [39]. These mutations provide information that is useful for resolving evolutionary relationships in phylogenetic analyses of related taxa [40]. We detected 44 indels among *A*. *lancea*, *A*. *chinensis*, *and A*. *macrocephala* (S8 Table), in which 37, 3, and 4 are located in the IGS, intronic regions, and CDS, respectively. Most indels range from 1 bp to 6 bps in length, and seven indels are longer than 10 bps. The longest indel with the length of 24 bps was found in the CDS of psbN. We also detected 111 single nucleotide diversity sites in the plant species (S9 Table), 42 of which are located in the CDS. In particular, the *ycf*1 gene contains 10 variation sites, thereby representing a variation hotspot.

### Comparative analyses on the three plastomes

**Fig 2:**
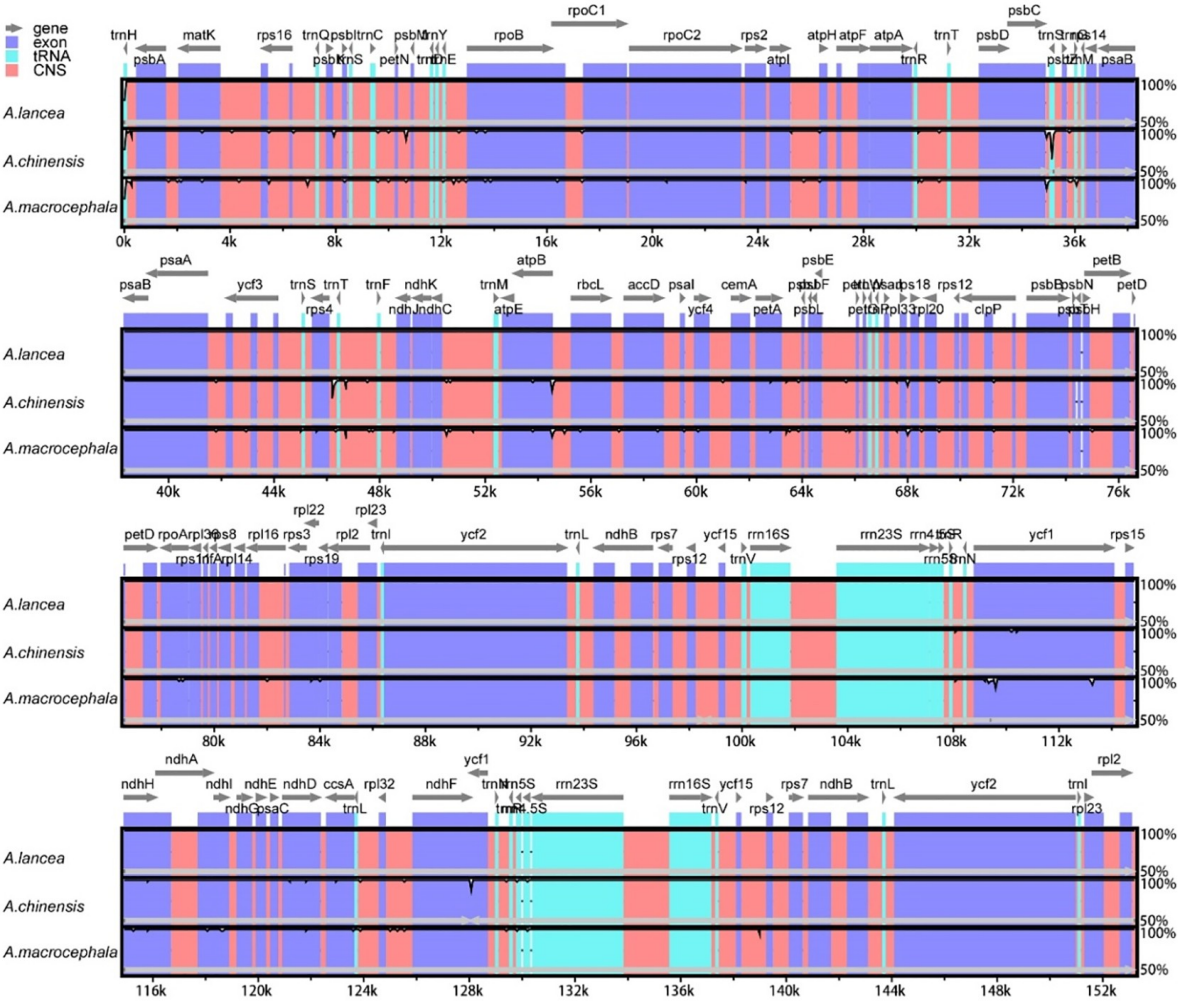
Sequence identity plot comparing the three plastomes with *A*. *lancea* as the reference by using mVISTA. Gray arrows above the alignment indicate genes and their orientation, with their names labeled on top of the arrows. A cut-off of 70% identity was used to make the plots. The x-axis indicates the position of the plastomes, and the y-axis represents the percent identity ranging from 50% to 100%. Regions colored differently represent gene, exon, tRNA, and CNS. CNS: conserved noncoding sequences.

The plastomes of *A*. *lancea*, *A*. *chinensis*, *and A*. *macrocephala* were compared and identified divergent regions (Fig 2). The *A*. *lancea* plastome serves as the reference. The genome organizations and sequences from the three plastomes are highly conserved and similar. Results revealed that the IR regions are more highly conserved than the LSC and SSC regions, and the coding regions are more conservative than the noncoding counterparts. We also found that the most divergent coding regions in the three plastomes are *rpb*B and *ycf*1 genes. Moreover, many noncoding regions, such as *psb*C-*psb*Z, *psb*Z*-rps*14, *rps*4-*trn*F, and *atp*B-*rbc*L, show high degree of sequence divergence.

### Phylogenetic analyses using plastome data

**Fig 3:**
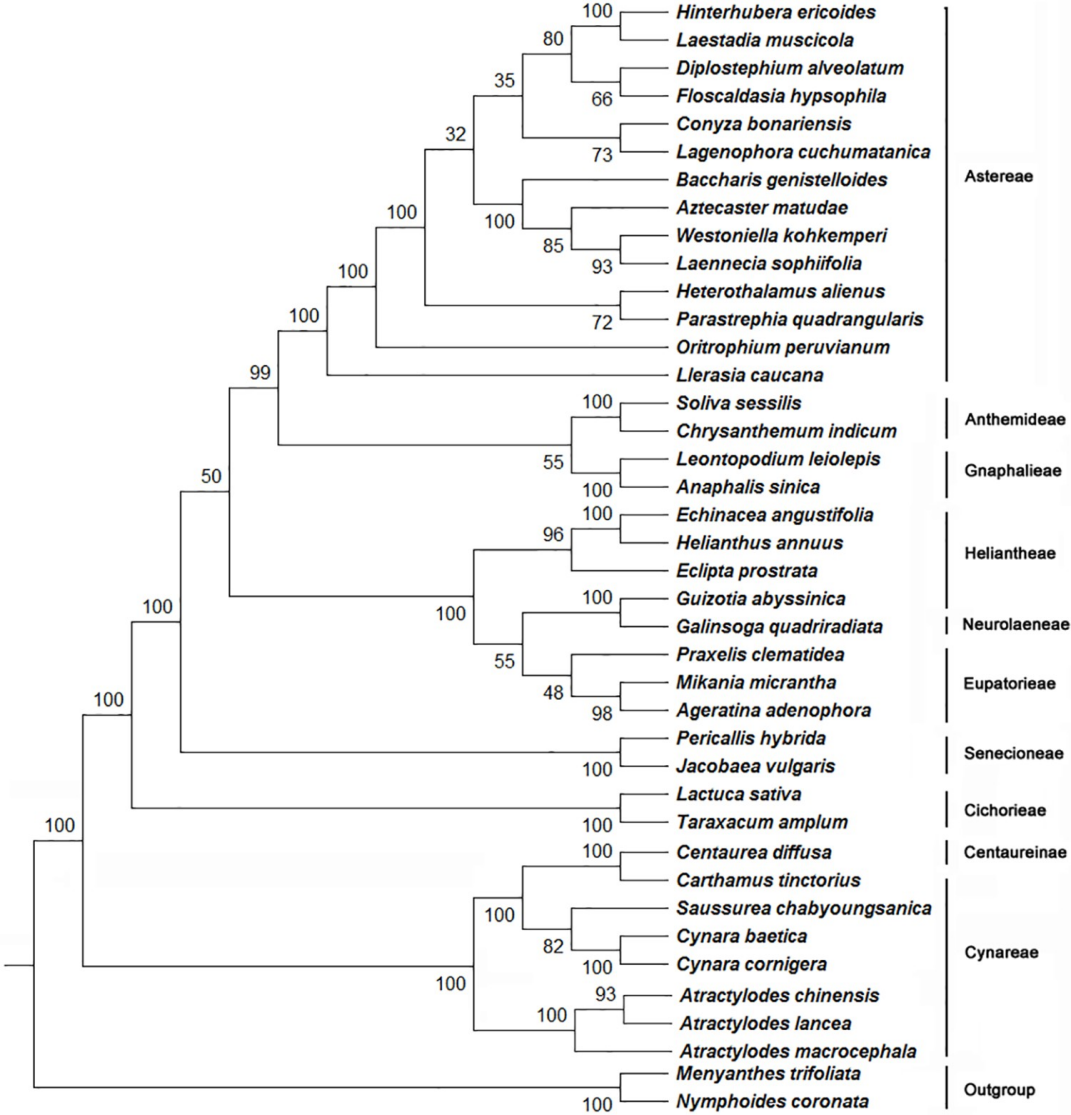
Molecular phylogenetic analyses. Plastome sequences of 64 common proteins present from 40 species (*Ageratina adenophora*, *Anaphalis sinica*, *Atractylodes chinensis*, *Atractylodes lancea*, *Atractylodes macrocephal*, *Aztecaster matudae*, *Baccharis genistelloides*, *Carthamus tinctorius*, *Centaurea diffusa*, *Chrysanthemum indicum*, *Conyza bonariensis*, *Cynara baetica*, *Cynara cornigera*, *Diplostephium alveolatum*, *Echinacea angustifolia*, *Eclipta prostrata*, *Floscaldasia hypsophila*, *Galinsoga quadriradiata*, *Guizotia abyssinica*, *Helianthus annuus*, *Heterothalamus alienus*, *Hinterhubera ericoides*, *Jacobaea vulgaris*, *Lactuca sativa*, *Laennecia sophiifolia*, *Laestadia muscicola*, *Lagenophora cuchumatanica*, *Leontopodium leiolepis*, *Llerasia caucana*, *Menyanthes trifoliate*, *Mikania micrantha*, *Oritrophium peruvianum*, *Parastrephia quadrangularis*, *Pericallis hybrida*, *Praxelis clematidea*, *Saussurea chabyoungsanica*, *Scaevola taccada*, *Soliva sessilis*, *Taraxacum amplum*, and *Westoniella kohkemperi*) were used to construct the phylogenetic tree with the maximum likelihood method implemented in the RAxML. Two taxa, namely, *Menyanthes trifoliata* and *Nymphoides coronata*, which were the closest relatives based on the APG IV system, were used as outgroups. Tribes to which each species belongs are shown on the right side of the tree. Bootstrap supports were calculated from 1000 replicates.

Phylogenetic relationships within the order of Asterales have been resolved in recent published reports. However, the position of *Atractylodes* in the family of Asteraceae remains controversial [4]. To determine the phylogenetic relationship of *A*. *lancea*, *A*. *chinensis*, and *A*. *macrocephala*, the plastomes of 35 other Asteraceae species and those of *Nymphoides coronata* and *Menyanthes trifoliata* from Menyanthaceae were downloaded from GenBank (S2 Table). A total of 64 common protein-coding sequences were identified and used to establish a single alignment data matrix with 15,744 characters [15, 41, 42]. A phylogenetic tree was constructed using the maximum likelihood method (Fig 3). In general, all the 38 species form a lineage (Asteraceae) that is recognizably discrete from the outgroup species of *Nymphoides coronata* and *Menyanthes trifoliata* from Menyanthaceae. These 38 species are grouped into 12 clades. Almost all the nodes in the phylogenetic tree show a strong bootstrap support. The three *Atractylodes* species are under Cynareae, which is grouped together with Centaureinae as a clade with a strong support. By contrast, Heliantheae, Neurolaeneae, and Eupatorieae are grouped together into another clade. These results rebuilt the phylogenetic relationship of *Atractylodes* species at the subfamily level.

### Development and validation of the molecular markers for species authentication

**Fig 4:**
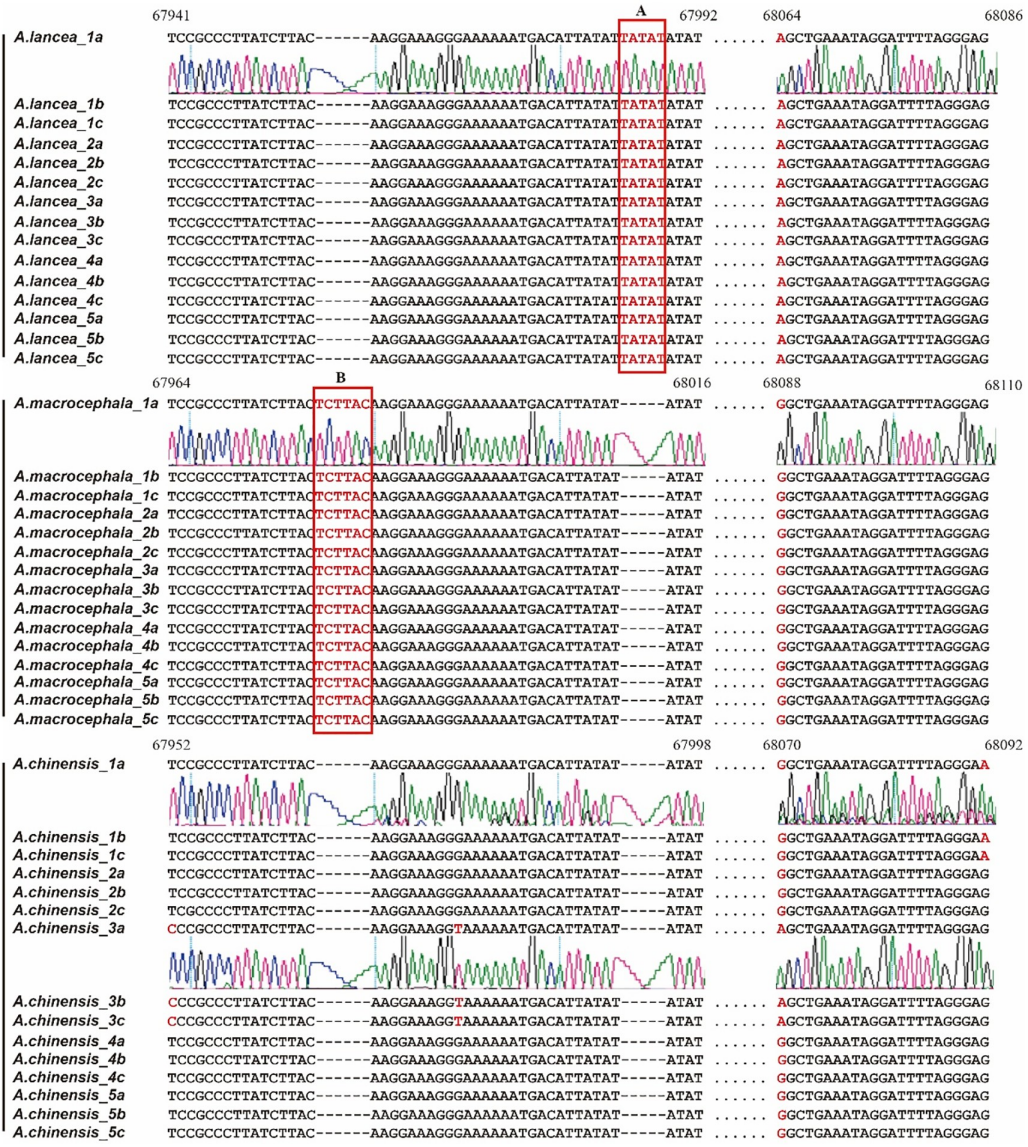
Alignment of the cz11 sequences from 15 individual plant samples of the three *Atractylodes* species. Arabic numerals represent different individuals. Letters a, b, and c represent duplicated Sanger sequencing results, and the red bases indicate different bases among the three species. The two regions (A and B) that can be used to distinguish the three species are highlighted with squares.

Using the seqDivGap algorithm, eleven regions were selected for further analyses. Primer pairs were designed for each region. Five plant individuals from each species were collected (S1 Table) and subjected to DNA extraction. The primer pairs were used to amplify the extracted plastome DNA from each individual plant, and the products were sent for Sanger sequencing. All 15 PCR products derived from the plant species were amplified and sequenced with the seven primer sets. However, only the primer pair cz11 produced good results, which was designed from region 6. The corresponding products were named as marker cz11 and were sequenced three times from each direction at least once to obtain high-quality sequencing results. The alignment of the resulting 45 sequences of the PCR products is displayed in Fig 4.

The sequences of the three species are clearly distinguished from each other: *A*. *lancea* shows a 5 bps insertion sequence of TATAT compared with that of *A*. *chinensis* (square A), and *A*. *macrocephala* presents a 6 bps insertion sequence of TCTTAC compared with that of *A*. *chinensis* (square B). Notably, the sequences of each five samples of *A*. *lancea* and *A*. *macrocephala* are the same results for the primer set of cz11, and sequence of *A*. *chinensis* present three different patterns. The PCR products for *A*. *chinensis*_1 show a single nucleotide mutation of G to A (shown red), and the PCR products for *A*. *chinensis*_3 exhibit three single nucleotide mutations from T to C, G to T, and G to A (shown red). Taken together, these findings confirmed that the identified marker (called cz11 marker) can be used to distinguish the three *Atractylodes* species from one to another. Hence, seqDivGap is a powerful tool in selecting the regions to isolate such markers.

To further validate the reliability of the marker cz11 to distiguish the three species, we collected plant materials from at least 3 origins for each species. At least 6 individuals were collected from each of three origins for each species (Table 3). For A. lancea, plants materials were further collected from 6, 7 and 9 individuals from three regions. All 76 individuals were subjected DNA extraction, PCR amplification with primer pair cz11 and Sanger sequencing as described above. The alignment of the 76 sequences of the PCR products is displayed in S3 Fig. For *A*. *lancea*, only 4 individuals from two geographic origins were validated successfully. The rest of them showed the same pattern as those of the *A*. *chinensis*. It is possible that the *A*. *lancea* samples were already mixed up with *A*. *chinensis*. In contrast, all 36 individuals from the *A*. *chinensis* and *A*. *macrocephala* were validated respectively, being found to have the expected marker sequences (Table 3).

**Table 3:** The information for the samples collected from the field and their validation results.

No.	Species Claimed	Origins of field test samples collected from China	Total number of individuals	Number of individuals having the expected marker
**1**	*A*. *lancea*	Ankang City, Shanxi, China	18	2
**2**	*A*. *lancea*	Suizhou City, Hubei, China	6	0
**3**	*A*. *lancea*	Harbin City, Heilongjiang, China	7	2
**4**	*A*. *lancea*	Bozhou City, Anhui, China	9	0
**5**	*A*. *macrocephala*	Bozhou City, Anhui, China	6	6
**6**	*A*. *macrocephala*	Enshi City, Hubei, China	6	6
**7**	*A*. *macrocephala*	Luohe City, Henan, China	6	6
**8**	*A*. *chinensis*	Luoyang City, Henan, China	6	6
**9**	*A*. *chinensis*	Fushun City, Liaoning, China	6	6
**10**	*A*. *chinensis*	Chifeng City, Inner Mongolia, China	6	6

### Allele variation profile analyses

The Sanger sequencing results show considerably limited depth. To further understand the degree of diversity in the cz11 marker locus, the NGS data were thus used to obtain the plastomes. The NGS data were generated from mixed DNAs derived from the five individual plants. We extracted the reads covering the cz11 marker sequences with BLAST. They were subsequently mapped to the reference plastome sequences using Bowtie2. The mapping results are shown in Table 4. In total, 445, 620, and 762 reads were mapped to the sequences of *A*. *lancea*, *A*. *macrocephala*, and *A*. *chinensis*, respectively. The sequence '------AAAGAAAGGGAAAAAATGACATTATATTATAT' shows the highest frequency in *A*. *lancea*, and ' TCTTACAAGGAAAGGGAAAAAATGACATTATAT-----' displays the highest frequency in *A*. *macrocephala*. These two sequences are identical to those obtained from Sanger sequencing results. The two major types of sequences in *A*. *chinensis*, namely, ' ------AAAGAAAGGTAAAAAATGACATTATAT-----' and ' ------AAGGAAAGGGAAAAAATGACATTATAT-----', exhibit comparable frequencies. Both patterns were also observed in the Sanger sequencing results.

**Table 4:** Types and frequencies of individual allele sequence of the cz11 marker loci among the three *Atractylodes* species.

Species	Sequence Type	Frequency
***A*. *lancea***	------AAGGAAAGGGAAAAAATGACATTATATTATAT	356
	------AAGGAAAGGGAAAAAATGACATTATAT-----	8
	------AAGGAAAAGGAAAAAATGACATTATATTATAT	3
	------AAGGAAAGAGAAAAAATGACATTATATTATAT	3
	------AAGGAAAGGGAAAAAAAGACATTATATTATAT	3
	------AAAGAAAGGGAAAAAATGACATTATATTATAT	2
	------AAGGAAAGGAAAAAAATGACATTATATTATAT	2
	------AAGGAAAGGGAAAAAAGGACATTATATTATAT	2
	------AAGGAAAGGGAAAAAATGACATTATAATATAT	2
	------AAGGAAAGGGAAAAAATGACATTATA-----T	2
	------AAGGAAAGGGAAAAAATGACATTATATTAT--	2
	------AAGGAAAGGGAAAAAATGACATTATATTATCT	2
	------AAGGAAAGGGAAAAAATGACCTTATATTATAT	2
	------AAGGAAAGGGAAACAATGACATTATATTATAT	2
	------AAGGCAAGGGAAAAAATGACATTATATTATAT	2
	------AAGGTAAGGGAAAAAATGACATTATATTATAT	2
	------AATGAAAGGGAAAAAATGACATTATATTATAT	2
	------CAGGAAAGGGAAAAAATGACATTATATTATAT	2
***A*. *macrocephala***	TCTTACAAGGAAAGGGAAAAAATGACATTATAT-----	588
	TCTTAAAAGGAAAGGGAAAAAATGACATTATAT-----	2
	TCTTACAAGGAAAGAGAAAAAATGACATTATAT-----	2
	TCTTACAAGGAAAGGGAAAAAATTACATTATAT-----	2
***A*. *chinensis***	------AAGGAAAGGTAAAAAATGACATTATAT-----	420
	------AAGGAAAGGGAAAAAATGACATTATAT-----	309
	------AAGGAAAGGGAAAAAAAGACATTATAT-----	3
	------AAGGAAAGGGAAAAA-TGACATTATAT-----	3
	------AAGGAAAGAGAAAAAATGACATTATAT-----	2
	------AAGGAAAGATAAAAAATGACATTATAT-----	2

With regard to frequencies, the dominant alleles of *A*. *lancea* and *A*. *macrocephala* represent 80% (356/445) and 94.84% (588/620) of all NGS reads mapped to the locus, respectively. The two dominant alleles found in *A*. *chinensis* represent 40.55% (309/762) and 55.12% (420/762) of the total reads mapped. In Sanger sequencing results, the sequence frequencies for the dominant alleles in *A*. *lancea* and *A*. *macrocephala* are both 100% (15/15). The frequencies of the two dominant alleles in *A*. *chinensis* are 80% (12/15) and 20% (3/15). This result suggested that the sequences obtained using the PCR and Sanger sequencing methods are biased, and they tend to lose the nucleotide diversity.

### Relationship of the three *Atractylodes* species using nuclear genes

**Fig 5:**
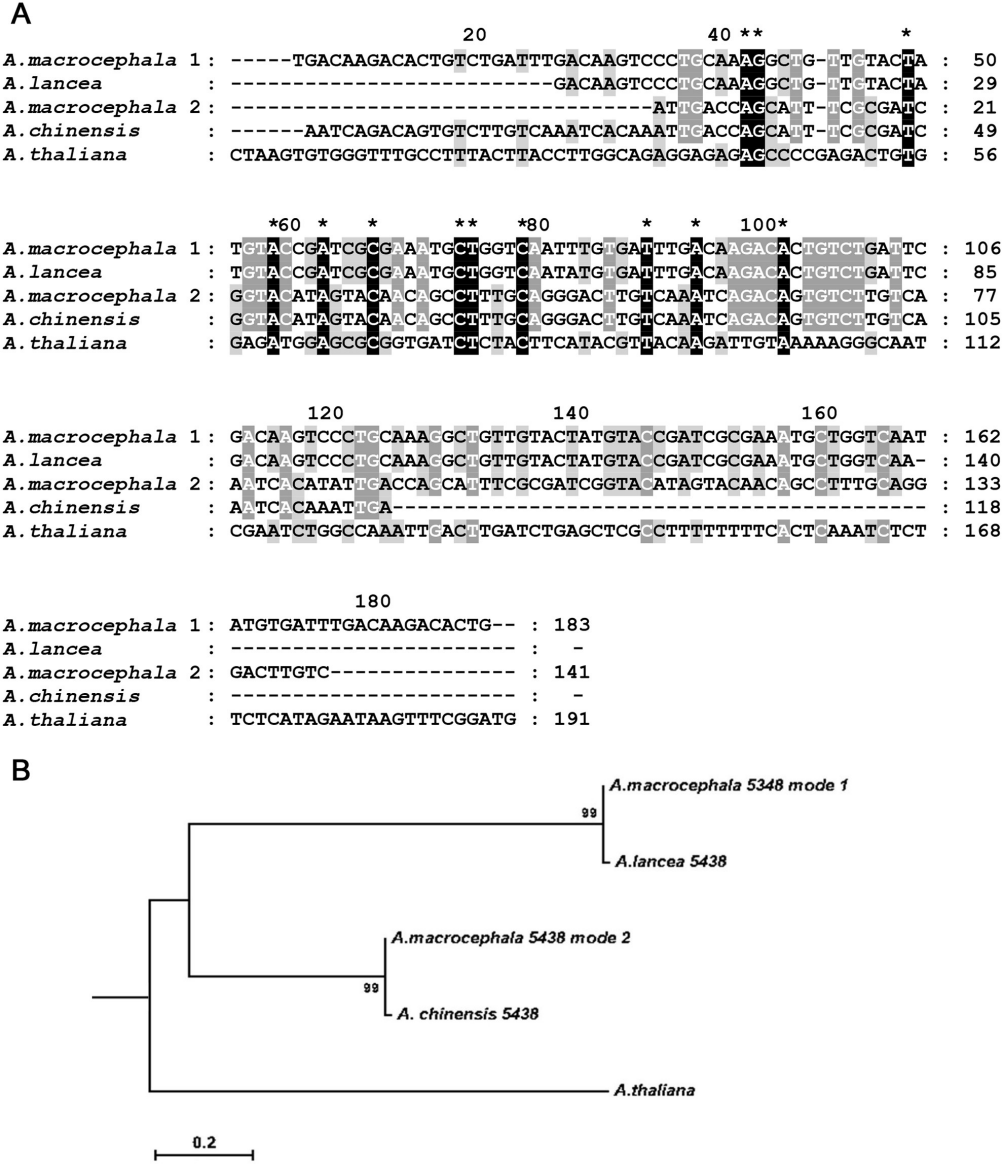
Multiple sequence alignment and phylogenetic analysis of nuclear gene SLD5 from three *Atractylodes* species. (A) Alignment of two sequences from *A*. *macrocephala* and one sequence from *A*. *lancea* and *A*. *chinensis* each. The sequence from *Arabidopsis thaliana* was provided as outer group. (B) Phylogenetic tree was constructed with the maximum likelihood method implemented in the RAxML. The *A*. *thaliana*, was used as the outgroup. Bootstrap supports were calculated from 1000 replicates.

Plastids are materinally inherited for most plants. It can not be used to determine the hybridization relationship among species. To overcome this limitation, we used the HybPiper software to compare the relationship of the three species based on 353 genes. Although reads were found mapping to all 353 genes, contigs were formed for 31, 30 and 24 genes in *A*. *lancea*, *A*. *macrocephala*, and *A*. *chinensis*, separately. In particular, contigs were found for 10 genes in all three species. Among them, and one gene named SLD5 (AT5G49010), had two contigs in *A*. *macrocephala*, and one contig in *A*. *lancea*, and *A*. *chinensis*, separately. SLD5 is a component of the heterotetrameric GINS complex and the GINS complex is essential for both the initiation and elongation stages of eukaryotic DNA replication. Then, multiple sequence alignment was performed for the four contigs in three species, using those sequences from *A*. *thaliana* as the outgroup (Fig 5A). Subsequently, phylogenetic analyses were performed with the alignment. Surprisingly, one contig in *A*. *macrocephala* was grouped with that of *A*. *lancea*, and the other contig of *A*. *macrocephala* was grouped with the contig i*n A*. *chinensis* (Fig 5B). It suggests that *A*. *macrocephala* may be a hybrid species of *A*. *lancea* and *A*. *chinensis*.

## Discussion

In this study, we (1) sequenced the plastomes for three *Atractylodes* species using the next-generation DNA sequencing technology; (2) annotated the plastomes; (3) identified indels, SSRs, tandem repeats, and SNPs of plastomes; (4) carried out a phylogenetic analysis of 40 related plastomes based on 64 conserved proteins; (5) identified 11 regions that potentially harbor good molecular markers; and (6) experimentally validated that top-ranked region harbors molecular markers; (7) clarify the relationship of the three species using nuclear genes. Our results provide a basis for future studies on the evolution of plastomes from *Atractylodes* species. In addition, a marker was identified to differentiate the closely related *Atractylodes* species. This study also discovered markers from less variable hyperplasmic regions. And for the first time, we report that *A*. *macrocephala* maybe a hybrid species of *A*. *lancea* and *A*. *chinensis*. However, this conclusion is rather weak and additional data are needed to confirm if this hypothesis is correct. If this is confirmed, it would explain the difficulty in distinguishing these species by chemical components and DNA barcodes in previous studies.

Our results contributed to the phylogenetic classification of *Atractylodes* species. Previous reports have established the phylogenetic relationship of these three *Atractylodes* species based on *trn*L-F sequences and ITS region sequences. The two trees are incongruent [1, 2, 4]. In the present study, we used 64 protein sequences to construct the phylogenetic trees, which contain many informative sites and generate a highly congruent tree.

The marker identified in this study for *Atractylodes* species differentiation is superior to previous markers, such as the *trn*K coding region and ITS region in several aspects. In the *trn*K coding region, the sequences of *A*. *lancea* and *A*. *chinensis* only differ at ployA sites. The number of nucleotides in the polyA stretch in the plastome is unstable in the same species [1,3]. Moreover, the PCR product of *trn*K gene is 2.6 kbs, which is considerably too long to be sequenced entirely using the Sanger method at a time. In the ITS regions, the differentiation of the three *Atractylodes* species depends on the substitution of seven nucleotide sites. The ITS regions are multiallelic. The differences of these substitution sites at intraspecific levels are unclear. Consequently, the use of ITS sequences for species determination is questionable. Taken together, identifying *Atractylodes* species by *trn*K or ITS sequences at the species and intraspecific levels is difficult [3]. We also systematically analyzed inter- and intraspecific nucleotide diversities of the cz11 markers based on the results obtained from Sanger sequencing and the NGS technologies. We also validated the identified markers for five individuals from each of the three species and confirmed the effectiveness of the cz11 marker. To further validate the reliability of cz11 marker, different individuals from different origins were validated. The cz11 marker can distinguish the *A*. *chinensis* and *A*. *macrocephala* species very reliably. However, using this marker, only 4 out of 40 claimed *A*. *lancea* samples were identified as *A*. *lancea*, while the other 36 were identifed as *A*. *chinensis*. Considering that the voucher samples from the garden of IMPLAD have been identified by experts. The most likely explanation is that some of the “*A*. *lancea*” samples from the market had already been “contaminated” with *A*. *chinensis*. Additional studies are needed to further clarify this issue. The cz11 marker could be of higher value for identifying the authenticity of medicinal materials of *Atractylodes*.

Previously, we showed the presence of intraspecific and heterplasmic variable regions in the plastome [43]. With the continuous influx of deep-sequencing data, heteroplasmicity should be the rule over the exception in the plastomes by considering the presence of multiple plastids in any given cell. Whether these heteroplasmic variable regions can be used for differentiating closely related species at the species or subspecies levels is also unclear. In this study, we initially designed 12 primer pairs manually by determining the variable regions among the consensus sequences of the three plastomes. Nevertheless, 11 out of the 12 primers generate no successful sequences using Sanger sequencing technology. These interspecific variable regions also show a high degree of heteroplasmicity. Therefore, a correlation exists between inter- and intraspecific variabilities. The primer pairs essentially amplify a set of products with high degree of variations (e.g., variable length), which prevent the yield of unambiguous sequences. The ideal regions for marker discovery may be those more variable interspecifically and less variable intraspecifically.

We developed the seqDivGap algorithm to identify these kinds of regions. Essentially, the seqDivGap takes advantage of the NGS reads to calculate the intraspecific nucleotide diversity. Afterward, we ranked the regions based on large interspecific diversity, low intraspecific diversity, and large difference between inter- and intraspecific diversities. Our previous experience suggested that the presence of polynucleotide stretches may result in poor sequencing quality due to the variable length of these stretches. We subsequently included additional criteria into the seqDivGap to screen the regions with long polynucleotides. Experimental validation proved that the primer designed based on the top ranked regions can identify variable regions that can generate high-quality sequences.

There are several lines of evidence support the hypothesis that *A*. *macrocephala* be the hybrid of *A*. *lancea* and *A*. *chinensis*. Firstly, the hybridization phenomenon of *A*. *lancea* and *A*. *chinensis* was shown possible in a previous study [3]. Secondly, the distribution area of *A*. *macrocephala* was between those of the *A*. *lancea* and the *A*. *chinensis*, making the above hypothesis possbile geographically. Thirdly, in this study, we found one gene named SLD5 had two contigs in *A*. *macrocephala*. One was clustered together with a sequence of *A*. *lancea*, and the other was clustered together with a sequence of *A*. *chinensis* in the phylogenetic tree, suggesting that *A*. *macrocephala* may be a hybrid of the other two species. On the other hand, the support of this hypothesis is relatively weak. Firstly, the monophyly and position of *A*. *macrocephala* described in previous phylogenetic studies suggested that it was not likely a hybrid of *A*. *lancea* and the *A*. *chinensis* [44, 45]. Secondly, only one gene was found to demonstate this pattern of hybridization in the current study. Taking together, this hypothesis needed to be tested in the future using data of higher sequencing depth and larger numbers of single copy genes.

## Conclusions

The complete plastomes of three *Atractylodes* species from Asteraceae were assembled, annotated and analyzed. The gene content, gene order, genome structure, SSRs and long repeats, and codon usage are largely similar. We then resolved the phylogenetic relationships of the three species in the family of Asteraceae with complete plastome sequences and nuclear genes. Finally, we identified a marker based on interspecific and intraspecific nucleotide diversity gaps to distinguish the three species. The maker was validated with 100% success rate using voucher samples. The barcode can be used to distinguish the three *Atractylodes* species, which will be invaluable to ensure the correct usage of *Atractylodes* materials in health food and herbal drugs.

## Supporting information

S1 Table Plant samples collected from the botantical garden used for validation of the molecular markers isolated with the seqDivGap method. (DOCX)

S2 Table List of plastomes used in this study and their origins. (DOCX)

S3 Table Primer sequences used for distinguishing the three *Atractylodes* species. (DOCX)

S4 Table Lengths of introns and exons for the splitting genes in the plastomes of *A*. *chinensis*, *A*. *lancea*, and *A*. *macrocephala*. (DOCX)

S5 Table Codon usage of protein-coding genes in the plastomes of the three *Atractylodes* species. (DOCX)

S6 Table Repeat sequences in the plastomes of the three *Atractylodes* species. (DOCX)

S7 Table Details for the simple sequence repeats (SSR) in the three *Atractylodes* species. (DOCX)

S8 Table Details of indels in the plastomes of the three *Atractylodes* species. (DOCX)

S9 Table Variation sites found among the plastomes of the three *Atractylodes* species. (DOCX)

S1 Fig Codon contents of 20 amino acid and stop codons in all protein-coding genes of the plastomes of the three Atractylodes species. The x-axis shows the amino acids and their codons. The y-axis shows the RSCU values. The columns represent the amino acids of A. lancea, A. chinensis, and A. macrocephala (from left to right). Different codons are labeled using different colors.
(DOCX)

S2 Fig Lengths and number of repeat sequences found in the three plastomes. REPuter was used to identify the repeat sequences with length ≥ 30 bp and sequence identity ≥ 90% in the plastomes. The repeats were further binned according to their length. The x-axis shows the species, the type of repeat (F or P), and the bin of the repeats based on length. The y-axis shows the number of repeats in each bin. The numbers of repeats in each bin are also shown on the top of the corresponding columns. F: forward repeat; P: palindrome repeat; Green: repeat length in the range of 30–39; Red: repeat length in the range of 40–49; Yellow: repeat length in the range of 50–59.
(DOCX)

S3 Fig Validation of the marker cz11 using different individual plant samples of the three *Atractylodes* species collected from different origins. AL_AK: *A*. *lancea* collected from Ankang City, Shanxi Province. AL_SZ: *A*. *lancea* collected from Suizhou City, Hubei Province. AL_HB: *A*. *lancea* collected from Harbin City, Heilongjiang Province. AL_BZ: *A*. *lancea* collected from Bozhou City, Anhui Province. AM_BZ: *A*. *macrocephala* collected from Bozhou City, Anhui Province. AM_ES: *A*. *macrocephala* collected from Enshi City, Hubei Provincce. AM_LH: *A*. *macrocephala* collected from Luohe City, Henan Province. AC_LY: *A*. *chinensis* collected from Luoyang City, Henan Province. AM_BZ: *A*. *macrocephala* collected from Bozhou City, Anhui Province. AM_ES: A. macrocephala collected from Enshi City, Hubei Provincce. AM_LH: A. macrocephala collected from Luohe City, Henan Province. AC_LY: *A*. *chinensis* collected from Luoyang City, Henan Province. AC_FS: *A*. *chinensis* collected from Fushun City, Liaoning Province. AC_CF: *A*. *chinensis* collected from Chifeng City, Inner Mongolia.
(DOCX)
