# Supplementary material for: Phylogenetic relationships of Atractylodes lancea, A. chinensis and A. macrocephala, revealed by complete plastome and nuclear gene sequences
Source: PLoS One. 2020 Jan 28;15(1):e0227610. doi: 10.1371/journal.pone.0227610 (PMC6986703; doi:10.1371/journal.pone.0227610)
Supplement: S1 Table — (DOCX) [file pone.0227610.s001.docx]

**Table S1. Plant samples of *Atractylodes* used for validation of the molecular markers isolated with the seqDivGap method.**

| **No.** | **Species Name** | **Collecting Place** | **Voucher Number** | **Sample ID** |
| --- | --- | --- | --- | --- |
| **1** | *A. lancea* | IMPLAD^a^, Beijing, China | Implad-20170101 | *A. lancea*_01 |
| **2** | *A. lancea* | IMPLAD, Beijing, China | Implad-20170102 | *A. lancea*_02 |
| **3** | *A. lancea* | IMPLAD, Beijing, China | Implad-20170103 | *A. lancea*_03 |
| **4** | *A. lancea* | IMPLAD, Beijing, China | Implad-20170104 | *A. lancea*_04 |
| **5** | *A. lancea* | IMPLAD, Beijing, China | Implad-20170105 | *A. lancea*_05 |
| **6** | *A. chinensis* | IMPLAD, Beijing, China | Implad-20170106 | *A. chinensis*_01 |
| **7** | *A. chinensis* | IMPLAD, Beijing, China | Implad-20170107 | *A. chinensis*_02 |
| **8** | *A. chinensis* | IMPLAD, Beijing, China | Implad-20170108 | *A. chinensis*_03 |
| **9** | *A. chinensis* | IMPLAD, Beijing, China | Implad-20170109 | *A. chinensis*_04 |
| **10** | *A. chinensis* | IMPLAD, Beijing, China | Implad-20170110 | *A. chinensis*_05 |
| **11** | *A. macrocephala* | IMPLAD, Beijing, China | Implad-20170111 | *A. macrocephala*_01 |
| **12** | *A. macrocephala* | IMPLAD, Beijing, China | Implad-20170112 | *A. macrocephala*_02 |
| **13** | *A. macrocephala* | IMPLAD, Beijing, China | Implad-20170113 | *A. macrocephala*_03 |
| **14** | *A. macrocephala* | IMPLAD, Beijing, China | Implad-20170114 | *A. macrocephala*_04 |
| **15** | *A. macrocephala* | IMPLAD, Beijing, China | Implad-20170115 | *A. macrocephala*_05 |

a: Beijing Medicinal Plant Garden, Institute of Medicinal Plant Development (IMPLAD), Beijing, China
