# Supplementary material for: Phylogenetic relationships of Atractylodes lancea, A. chinensis and A. macrocephala, revealed by complete plastome and nuclear gene sequences
Source: PLoS One. 2020 Jan 28;15(1):e0227610. doi: 10.1371/journal.pone.0227610 (PMC6986703; doi:10.1371/journal.pone.0227610)
Supplement: S2 Table — (DOCX) [file pone.0227610.s002.docx]

**Table S2. List of plastomes used in this study and their origins*.***

| **No.** | **Species Name** | **NCBI Accession Number** | **Tribe** | **Subfamily** |
| --- | --- | --- | --- | --- |
| **1** | *Centaurea diffusa* | NC_024286/KJ690264 | Centaureinae | Asteraceae |
| **2** | *Chrysanthemum indicum* | NC_020320/JN867589 | Anthemideae | Asteroideae |
| **3** | *Soliva sessilis* | NC_034851/KX063863 | Anthemideae | Asteroideae |
| **4** | *Aztecaster matudae* | NC_034898/KX063935 | Astereae | Asteroideae |
| **5** | *Baccharis genistelloides* | NC_034852/KX063864 | Astereae | Asteroideae |
| **6** | *Conyza bonariensis* | NC_035884/MF276802 | Astereae | Asteroideae |
| **7** | *Diplostephium alveolatum* | NC_034847/KX063856 | Astereae | Asteroideae |
| **8** | *Floscaldasia hypsophila* | NC_034888/KX063916 | Astereae | Asteroideae |
| **9** | *Heterothalamus alienus* | NC_034855/KX063869 | Astereae | Asteroideae |
| **10** | *Laennecia sophiifolia* | NC_034877/KX063899 | Astereae | Asteroideae |
| **11** | *Laestadia muscicola* | NC_034858/KX063873 | Astereae | Asteroideae |
| **12** | *Lagenophora cuchumatanica* | NC_034819/KX063879 | Astereae | Asteroideae |
| **13** | *Oritrophium peruvianum* | NC_034849/KX063861 | Astereae | Asteroideae |
| **14** | *Parastrephia quadrangularis* | NC_034890/KX063923 | Astereae | Asteroideae |
| **15** | *Westoniella kohkemperi* | NC_034889/KX063921 | Astereae | Asteroideae |
| **16** | *Llerasia caucana* | NC_034821/KX063908 | Astereae | Asteroideae |
| **17** | *Ageratina adenophora* | NC_015621/JF826503 | [Eupatorieae](https://en.wikipedia.org/wiki/Eupatorieae) | Asteroideae |
| **18** | *Mikania micrantha* | NC_031833/KX154571 | Eupatorieae | Asteroideae |
| **19** | *Praxelis clematidea* | NC_023833/KF922320 | Eupatorieae | Asteroideae |
| **20** | *Anaphalis sinica* | NC_034648/KX148081 | Gnaphalieae | Asteroideae |
| **21** | *Leontopodium leiolepis* | NC_027835/KM267636 | Gnaphalieae | Asteroideae |
| **22** | *Echinacea angustifolia* | NC_034324 | Heliantheae | Asteroideae |
| **23** | *Eclipta prostrata* | NC_030773/KU361242 | Heliantheae | Asteroideae |
| **24** | *Guizotia abyssinica* | NC_010601/EU549769 | Heliantheae | Asteroideae |
| **25** | *Helianthus annuus* | NC_007977/DQ383815 | Heliantheae | Asteroideae |
| **26** | *Galinsoga quadriradiata* | NC_031853/KX752097 | Neurolaeneae | Asteroideae |
| **27** | *Jacobaea vulgaris* | NC_015543/HQ234669 | Senecioneae | Asteroideae |
| **28** | *Pericallis hybrida* | NC_031898/KT285537 | Senecioneae | Asteroideae |
| **29** | *Carthamus tinctorius* | NC_030783/KM207677 | Cynareae | Carduoideae |
| **30** | *Cynara baetica* | NC_028005/KP842706 | Cynareae | Carduoideae |
| **31** | *Cynara cornigera* | NC_028006.1/KP842707.1 | Cynareae | Carduoideae |
| **32** | *Saussurea chabyoungsanica* | NC_036677/KX622799 | Cynareae | Carduoideae |
| **33** | *Atractylodes lancea* | MG874804 | Cynareae | Carduoideae |
| **34** | *Atractylodes chinensis* | MG874805 | Cynareae | Carduoideae |
| **35** | *Atractylodes macrocephala* | MN661162 | Cynareae | Carduoideae |
| **36** | *Lactuca sativa* | NC_007578/AP007232 | Cichorieae | Cichorioideae |
| **37** | *Taraxacum amplum* | NC_031816/KX499525 | Cichorieae | Cichorioideae |
| **38** | *Hinterhubera ericoides* | NC_034884/KX063910 | Astereae | Asteroideae |
| **39** | *Menyanthes trifoliata* | NC_041436 | ^a^NA | ^a^NA |
| **40** | *Nymphoides coronata* | NC_041484 | ^a^NA | ^a^NA |

^a^NA: Not applicable
