# Supplementary material for: Phylogenetic relationships of Atractylodes lancea, A. chinensis and A. macrocephala, revealed by complete plastome and nuclear gene sequences
Source: PLoS One. 2020 Jan 28;15(1):e0227610. doi: 10.1371/journal.pone.0227610 (PMC6986703; doi:10.1371/journal.pone.0227610)
Supplement: S3 Table — (DOCX) [file pone.0227610.s003.docx]

**Table S3. Primer sequences used for distinguishing the three *Atractylodes* species.**

| **Primer ID** | **Primer Sequence** | **Primer Position** | | | | | |
| --- | --- | --- | --- | --- | --- | --- | --- |
|  |  | ***A. lancea*** | | ***A. chinensis*** | | ***A. macrocephala*** | |
| **cz2_F** | CCGGTCCTTTGCTAATCGGA | 50218 | 50237 | 50214 | 50233 | 50220 | 20239 |
| **cz2_R** | ACAAGAGGATTTGGATAAGAGCA | 50549 | 50571 | 50543 | 50565 | 50545 | 50567 |
| **cz3_F** | ACTCGCGGATTTATCGACATCA | 63186 | 63207 | 63197 | 63218 | 63221 | 63232 |
| **cz3_R** | AGACGAATAACCCCTCCCAGA | 63558 | 63578 | 63568 | 63588 | 63580 | 63600 |
| **cz4_F** | TGCTACTCGAACCACGCTTT | 80518 | 80537 | 80550 | 50569 | 80544 | 80563 |
| **cz4_R** | GCGATTGCCCGAGAATTGAG | 80843 | 80862 | 80876 | 80895 | 80874 | 80893 |
| **cz5_F** | TGGGTCCTACGTCAATTCCAT | 10574 | 10594 | 10561 | 10581 | 10574 | 10594 |
| **cz5_R** | ACGGGCTATAATCGGCGGTA | 10752 | 10771 | 10730 | 10749 | 10752 | 10771 |
| **cz6_F** | TGCTTGGTCTTCACTGGAAACT | 17232 | 17253 | 17211 | 17232 | 17222 | 17243 |
| **cz6_R** | AATGTATCGAAGCTCCGGGT | 17505 | 17524 | 17484 | 17503 | 17496 | 17515 |
| **cz7_F** | AGGGTACTCAAGCGTTTCGG | 29896 | 29915 | 29871 | 29890 | 29884 | 29903 |
| **cz7_R** | CAAGCTAACGATGCGGGTTC | 30280 | 30299 | 30253 | 30272 | 30267 | 30286 |
| **cz8_F** | GGAGTCGCTTCAGTTCCCTC | 34912 | 34931 | 34885 | 34904 | 34899 | 34918 |
| **cz8_R** | GGTTTTGGCCGGATGGTTTC | 35060 | 35079 | 35041 | 35060 | 35055 | 35074 |
| **cz9_F** | TGGCGAAATTGGTAGACGCT | 47040 | 47059 | 47036 | 47055 | 47041 | 47060 |
| **cz9_R** | CCAACGTAAGACAATCAACCCC | 47252 | 47273 | 47248 | 47269 | 47253 | 47274 |
| **cz10_F** | CCTGGCGGAAAAGCTACATC | 54411 | 54430 | 54406 | 54425 | 54412 | 54431 |
| **cz10_R** | TAACCGATCAACTTGCCATCG | 54586 | 54606 | 54590 | 54610 | 54595 | 54615 |
| **cz11_F** | AGAACCGTCACAATACGCCT | 67834 | 67853 | 67845 | 67864 | 67857 | 67876 |
| **cz11_R** | AACGCCTACGAAAAGATCGC | 68143 | 68162 | 68149 | 68168 | 68167 | 68186 |
| **cz12_F** | TCTGTTAAAGGATACGTGGTTTGA | 125251 | 125274 | 125284 | 125307 | 125303 | 125326 |
| **cz12_R** | TCTGAGTCTTTTCAAAATCGTTCA | 125674 | 125697 | 125708 | 125731 | 125728 | 125751 |
