# Supplementary material for: Phylogenetic relationships of Atractylodes lancea, A. chinensis and A. macrocephala, revealed by complete plastome and nuclear gene sequences
Source: PLoS One. 2020 Jan 28;15(1):e0227610. doi: 10.1371/journal.pone.0227610 (PMC6986703; doi:10.1371/journal.pone.0227610)
Supplement: S4 Table — (DOCX) [file pone.0227610.s004.docx]

**Table S4. Lengths of introns and exons for the splitting genes in the plastomes of *A. chinensis,* *A. lancea*, and *A. macrocephala*.**

| **Gene** | **Strand** | **Region** | **Exon I** | **Intron I** | **Exon II** | **Intron II** | **Exon III** |
| --- | --- | --- | --- | --- | --- | --- | --- |
| ***trn*K-UUU** | − | LSC | 39 | 2555 | 36 |  |  |
| ***rps*16** | − | LSC | 45 | 846 | 222 |  |  |
| ***rpo*C1** | + | LSC | 432 | 740 | 1638 |  |  |
| ***atp*F** | + | LSC | 144 | 707 | 411 |  |  |
| ***trn*S-CGA** | − | LSC | 32 | 687 | 60 |  |  |
| ***ycf*3** | − | LSC | 126 | 697 | 228 | 737 | 153 |
| ***trn*L-UAA** | + | LSC | 36 | 441 | 51 |  |  |
| ***trn*C-ACA** | − | LSC | 39 | 553 | 57 |  |  |
| ***clp*P** | − | LSC | 69 | 795 | 294 | 628 | 228 |
| ***pet*B** | + | LSC | 9 | 1030 | 639 |  |  |
| ***pet*D** | + | LSC | 9 | 826 | 474 |  |  |
| ***rpl*16** | − | LSC | 9 | 715 | 399 |  |  |
| ***rpl*2** | − | IR | 390 | 666 | 435 |  |  |
| ***ndh*B** | − | IR | 777 | 670 | 756 |  |  |
| ***trn*E-UUC** | + | IR | 33 | 946 | 41 |  |  |
| ***trn*A-UGC** | + | IR | 38 | 820 | 36 |  |  |
| ***ndh*A** | + | SSC | 553 | 1059 | 539 |  |  |
| ***trn*A-UCC** | − | IR | 38 | 820 | 36 |  |  |
| ***trn*E-UUC** | − | IR | 33 | 946 | 41 |  |  |
| ***ndh*B** | + | IR | 777 | 670 | 756 |  |  |
| ***rpl*2** | + | IR | 390 | 666 | 435 |  |  |

LSC: large single-copy region. SSC: small single-copy regions. IR: inverted repeat regions.
