# Supplementary material for: Phylogenetic relationships of Atractylodes lancea, A. chinensis and A. macrocephala, revealed by complete plastome and nuclear gene sequences
Source: PLoS One. 2020 Jan 28;15(1):e0227610. doi: 10.1371/journal.pone.0227610 (PMC6986703; doi:10.1371/journal.pone.0227610)
Supplement: S5 Table — (DOCX) [file pone.0227610.s005.docx]

**Table S5. Codon usage of protein-coding genes in the plastomes of the three *Atractylodes* species.**

| **Codon Type** | ***A. lancea*** | | ***A. chinensis*** | | ***A. macrocephala*** | |
| --- | --- | --- | --- | --- | --- | --- |
|  | **Number** | **RSCU** | **Number** | **RSCU** | **Number** | **RSCU** |
| **GCU-A** | 356 | 1.91 | 351 | 1.91 | 357 | 1.92 |
| **GCC-A** | 118 | 0.63 | 117 | 0.64 | 117 | 0.63 |
| **GCA-A** | 192 | 1.03 | 190 | 1.03 | 191 | 1.03 |
| **GCG-A** | 80 | 0.43 | 78 | 0.42 | 80 | 0.43 |
| **UGU-C** | 88 | 1.56 | 88 | 1.53 | 88 | 1.56 |
| **UGC-C** | 25 | 0.44 | 27 | 0.47 | 25 | 0.44 |
| **GAU-D** | 357 | 1.59 | 356 | 1.59 | 356 | 1.58 |
| **GAC-D** | 92 | 0.41 | 93 | 0.41 | 94 | 0.42 |
| **GAA-E** | 452 | 1.52 | 448 | 1.51 | 451 | 1.52 |
| **GAG-E** | 143 | 0.48 | 147 | 0.49 | 144 | 0.48 |
| **UUU-F** | 392 | 1.34 | 387 | 1.33 | 391 | 1.34 |
| **UUC-F** | 193 | 0.66 | 194 | 0.67 | 193 | 0.66 |
| **GGU-G** | 287 | 1.37 | 286 | 1.37 | 288 | 1.37 |
| **GGC-G** | 117 | 0.56 | 117 | 0.56 | 117 | 0.56 |
| **GGA-G** | 298 | 1.42 | 295 | 1.41 | 297 | 1.41 |
| **GGG-G** | 139 | 0.66 | 139 | 0.66 | 140 | 0.67 |
| **CAU-H** | 222 | 1.52 | 222 | 1.52 | 222 | 1.52 |
| **CAC-H** | 70 | 0.48 | 70 | 0.48 | 70 | 0.48 |
| **AUU-I** | 448 | 1.55 | 452 | 1.56 | 448 | 1.55 |
| **AUC-I** | 183 | 0.63 | 182 | 0.63 | 184 | 0.64 |
| **AUA-I** | 235 | 0.81 | 237 | 0.82 | 233 | 0.81 |
| **AAA-K** | 361 | 1.54 | 360 | 1.52 | 360 | 1.54 |
| **AAG-K** | 109 | 0.46 | 115 | 0.48 | 109 | 0.46 |
| **UUA-L** | 372 | 1.94 | 372 | 1.93 | 372 | 1.94 |
| **UUG-L** | 244 | 1.27 | 245 | 1.27 | 243 | 1.27 |
| **CUU-L** | 242 | 1.26 | 245 | 1.27 | 241 | 1.26 |
| **CUC-L** | 70 | 0.36 | 69 | 0.36 | 70 | 0.36 |
| **CUA-L** | 147 | 0.77 | 148 | 0.77 | 147 | 0.77 |
| **CUG-L** | 77 | 0.4 | 77 | 0.4 | 78 | 0.41 |
| **AUG-M** | 235 | 1 | 235 | 1 | 235 | 1 |
| **AAU-N** | 370 | 1.54 | 365 | 1.54 | 371 | 1.54 |
| **AAC-N** | 110 | 0.46 | 109 | 0.46 | 110 | 0.46 |
| **CCU-P** | 185 | 1.61 | 186 | 1.61 | 185 | 1.61 |
| **CCC-P** | 73 | 0.63 | 72 | 0.62 | 73 | 0.63 |
| **CCA-P** | 133 | 1.15 | 132 | 1.14 | 133 | 1.15 |
| **CCG-P** | 70 | 0.61 | 72 | 0.62 | 70 | 0.61 |
| **CAA-Q** | 316 | 1.52 | 318 | 1.51 | 318 | 1.52 |
| **CAG-Q** | 101 | 0.48 | 102 | 0.49 | 101 | 0.48 |
| **AGA-R** | 172 | 1.66 | 171 | 1.66 | 172 | 1.67 |
| **AGG-R** | 64 | 0.62 | 65 | 0.63 | 63 | 0.61 |
| **CGU-R** | 152 | 1.47 | 152 | 1.47 | 151 | 1.46 |
| **CGC-R** | 46 | 0.45 | 48 | 0.47 | 46 | 0.45 |
| **CGA-R** | 140 | 1.35 | 137 | 1.33 | 140 | 1.36 |
| **CGG-R** | 46 | 0.45 | 46 | 0.45 | 47 | 0.46 |
| **AGU-S** | 177 | 1.39 | 175 | 1.37 | 177 | 1.39 |
| **AGC-S** | 40 | 0.31 | 40 | 0.31 | 40 | 0.31 |
| **UCU-S** | 237 | 1.86 | 242 | 1.9 | 237 | 1.86 |
| **UCC-S** | 111 | 0.87 | 111 | 0.87 | 112 | 0.88 |
| **UCA-S** | 142 | 1.12 | 139 | 1.09 | 142 | 1.11 |
| **UCG-S** | 57 | 0.45 | 57 | 0.45 | 57 | 0.45 |
| **ACU-T** | 237 | 1.72 | 236 | 1.71 | 239 | 1.73 |
| **ACC-T** | 115 | 0.83 | 115 | 0.83 | 114 | 0.83 |
| **ACA-T** | 153 | 1.11 | 155 | 1.12 | 153 | 1.11 |
| **ACG-T** | 46 | 0.33 | 46 | 0.33 | 46 | 0.33 |
| **GUU-V** | 222 | 1.4 | 223 | 1.4 | 222 | 1.4 |
| **GUC-V** | 67 | 0.42 | 68 | 0.43 | 66 | 0.42 |
| **GUA-V** | 262 | 1.65 | 262 | 1.65 | 263 | 1.66 |
| **GUG-V** | 84 | 0.53 | 84 | 0.53 | 84 | 0.53 |
| **UGG-W** | 209 | 1 | 212 | 1 | 209 | 1 |
| **UAU-Y** | 332 | 1.62 | 323 | 1.61 | 330 | 1.62 |
| **UAC-Y** | 77 | 0.38 | 79 | 0.39 | 77 | 0.38 |
| **UAA-*** | 18 | 1.69 | 20 | 1.58 | 18 | 1.64 |
| **UAG-*** | 7 | 0.66 | 10 | 0.79 | 7 | 0.64 |
| **UGA-*** | 7 | 0.66 | 8 | 0.63 | 8 | 0.73 |

RSCU: the relative synonymous codon usage values.
