# Supplementary material for: Phylogenetic relationships of Atractylodes lancea, A. chinensis and A. macrocephala, revealed by complete plastome and nuclear gene sequences
Source: PLoS One. 2020 Jan 28;15(1):e0227610. doi: 10.1371/journal.pone.0227610 (PMC6986703; doi:10.1371/journal.pone.0227610)
Supplement: S6 Table — (DOCX) [file pone.0227610.s006.docx]

**Table S6. Repeat sequences in the plastomes of the three *Atractylodes* species.**

| **Repeat Number** | ***A*. *lancea*** | | | | ***A*. *chinensis*** | | | | ***A*. *macrocephala*** | | | |
| --- | --- | --- | --- | --- | --- | --- | --- | --- | --- | --- | --- | --- |
|  | **Type** | **Size** | **Start** | **End** | **Type** | **Size** | **Start** | **End** | **Type** | **Size** | **Start** | **End** |
| **1** | P | 54 | 46684 | 46684 |  |  |  |  | P | 52 | 46686 | 46686 |
| **2** | P | 48 | 74369 | 74369 | P | 48 | 74377 | 74377 | P | 48 | 74395 | 74395 |
| **3** | P | 42 | 10696 | 10696 | P | 42 | 10674 | 10674 | P | 42 | 10696 | 10696 |
| **4** | P | 42 | 43342 | 117621 | P | 42 | 43324 | 117654 | P | 42 | 43342 | 117673 |
| **5** | F | 50 | 110123 | 110168 | F | 50 | 110156 | 110201 | F | 50 | 110169 | 110214 |
| **6** | F | 48 | 113137 | 113167 | F | 47 | 113181 | 113211 | F | 47 | 113194 | 113224 |
| **7** | F | 47 | 113148 | 113178 |  |  |  |  |  |  |  |  |
| **8** | F | 41 | 43343 | 98244 | F | 41 | 43325 | 98277 | F | 41 | 43343 | 98275 |
| **9** | P | 41 | 43343 | 139166 | P | 41 | 43325 | 139223 | P | 41 | 43343 | 139226 |
| **10** | P | 41 | 98244 | 117621 | P | 41 | 98277 | 117654 | P | 41 | 98275 | 117673 |
| **11** |  |  |  |  | F | 41 | 113170 | 113200 | F | 41 | 113183 | 113213 |
| **12** | F | 41 | 117621 | 139166 | F | 41 | 117654 | 139223 | F | 41 | 117673 | 139226 |
| **13** | F | 34 | 89110 | 89131 | F | 34 | 89143 | 89164 | F | 34 | 89141 | 89162 |
| **14** | P | 34 | 89110 | 148286 | P | 34 | 89143 | 148343 | P | 34 | 89141 | 148346 |
| **15** | P | 34 | 89131 | 148307 | P | 34 | 89164 | 148364 | P | 34 | 89162 | 148367 |
| **16** | F | 34 | 148286 | 148307 | F | 34 | 148343 | 148364 | F | 34 | 148346 | 148367 |
| **17** | P | 30 | 8517 | 45107 | P | 30 | 8504 | 45089 | P | 30 | 8517 | 45107 |
| **18** | F | 36 | 41906 | 56777 | F | 36 | 41888 | 56781 | F | 36 | 41906 | 56802 |
| **19** | P | 33 | 111745 | 114156 | P | 33 | 111778 | 114189 | P | 33 | 111791 | 114208 |
| **20** |  |  |  |  |  |  |  |  | F | 30 | 113300 | 113330 |
| **21** | F | 35 | 43348 | 95202 | F | 35 | 43330 | 95235 | F | 35 | 43348 | 95233 |
| **22** | P | 35 | 43348 | 142214 | P | 35 | 43330 | 142271 | P | 35 | 43348 | 142274 |
| **23** | P | 35 | 95202 | 117622 | P | 35 | 95235 | 117655 | P | 35 | 95233 | 117674 |
| **24** | F | 35 | 117622 | 142214 | F | 35 | 117655 | 142271 | F | 35 | 117674 | 142274 |
| **25** |  |  |  |  |  |  |  |  | F | 32 | 109651 | 109660 |
| **26** | P | 31 | 378 | 415 | P | 30 | 375 | 405 | P | 31 | 371 | 408 |
| **27** | F | 30 | 107325 | 107357 | F | 30 | 107358 | 107390 | F | 30 | 107362 | 107394 |
| **28** | P | 30 | 107325 | 130064 | P | 30 | 107358 | 130121 | P | 30 | 107362 | 130118 |
| **29** | P | 30 | 107357 | 130096 | P | 30 | 107390 | 130153 | P | 30 | 107394 | 130150 |
| **30** | F | 30 | 130064 | 130096 | F | 30 | 130121 | 130153 | F | 30 | 130118 | 130150 |
| **31** | F | 32 | 8515 | 35114 | F | 32 | 8502 | 35095 | F | 32 | 8515 | 35109 |
| **32** | F | 31 | 12451 | 12484 | F | 31 | 12430 | 12463 |  |  |  |  |
| **33** | F | 31 | 148295 | 148316 | F | 31 | 148352 | 148373 | F | 31 | 148355 | 148376 |
| **34** | F | 30 | 35047 | 40905 | P | 30 | 35097 | 45089 | P | 30 | 35111 | 45107 |
| **35** | P | 30 | 35116 | 45107 | P | 30 | 35155 | 45027 | P | 30 | 35169 | 45045 |
| **36** | P | 30 | 35174 | 45045 |  |  |  |  |  |  |  |  |
| **37** | P | 30 | 43346 | 75715 | P | 30 | 43328 | 75747 | P | 30 | 43346 | 75741 |
| **38** | F | 30 | 75715 | 117629 | F | 30 | 75747 | 117662 | F | 30 | 75741 | 117681 |
| **39** | F | 30 | 91485 | 91503 | F | 30 | 91518 | 91536 | F | 30 | 91516 | 91534 |
| **40** | P | 30 | 91485 | 145918 | P | 30 | 91518 | 145975 | P | 30 | 91516 | 145978 |
| **41** | P | 30 | 91503 | 145936 | P | 30 | 91536 | 145993 | P | 30 | 91534 | 145996 |
| **42** | F | 30 | 145918 | 145936 | F | 30 | 145975 | 145993 | F | 30 | 145978 | 145996 |

F: forward repeats. P: palindromic repeats.
