# Supplementary material for: Phylogenetic relationships of Atractylodes lancea, A. chinensis and A. macrocephala, revealed by complete plastome and nuclear gene sequences
Source: PLoS One. 2020 Jan 28;15(1):e0227610. doi: 10.1371/journal.pone.0227610 (PMC6986703; doi:10.1371/journal.pone.0227610)
Supplement: S7 Table — (DOCX) [file pone.0227610.s007.docx]

**Table S7. Details for the simple sequence repeats (SSR) in the three *Atractylodes* species.**

| **No.** | **Location** | **Region** | **Motif** | ***A*. *lancea*** | | ***A*. *chinensis*** | | ***A*. *macrocephala*** | |
| --- | --- | --- | --- | --- | --- | --- | --- | --- | --- |
|  |  |  |  | **Repeat Number** | **Position** | **Repeat Number** | **Position** | **Repeat Number** | **Position** |
| **1** | *psb*A-*mat*K | IGS | T | 11 | 1982 | 11 | 1975 | NA | NA |
| **2** | *mat*K-*rps*16 | IGS | AAAT | 3 | 4547 | 3 | 4540 | 3 | 4540 |
| **3** | *rps*16 | Intron | C | 11 | 5456 | 10 | 5449 | NA | NA |
| **4** | *trn*E-*rpo*B | IGS | T | 10 | 12937 | 10 | 12916 | 11 | 12926 |
| **5** | *rpo*B | CDS | A | 10 | 13629 | 10 | 13608 | 10 | 13619 |
| **6** | *rpo*C1 | Intron | T | 13 | 17312 | 12 | 17291 | NA | NA |
| **7** | *rpo*C1 | CDS | A | 10 | 18683 | 10 | 18662 | 10 | 18674 |
| **8** | *rpo*C1 | CDS | TA | 5 | 18906 | 5 | 18885 | 5 | 18897 |
| **9** | *rpo*C2 | CDS | AT | 5 | 19921 | 5 | 19900 | 5 | 19912 |
| **10** | *rpo*C2-*rps*2 | IGS | T | 11 | 23525 | 11 | 23504 | 10 | 23516 |
| **11** | *atp*I-*atp*H | IGS | T | 10 | 25251 | 10 | 25230 | 10 | 25241 |
| **12** | *atp*I-*atp*H | IGS | A | 10 | 25404 | 10 | 25381 | 10 | 25394 |
| **13** | *atp*I-*atp*H | IGS | T | 11 | 26345 | 10 | 26322 | 10 | 26335 |
| **14** | *atp*F-*atp*A | IGS | T | 12 | 28285 | 11 | 28261 | 11 | 28274 |
| **15** | *trn*R-*psb*D | IGS | A | 10 | 30097 | NA | NA | NA | NA |
| **16** | *psb*C | CDS | TTC | 4 | 34767 | 4 | 34740 | 4 | 34754 |
| **17** | *psb*C-*trn*S | IGS | T | 14 | 34968 | 14 | 34941 | 15 | 34955 |
| **18** | *psb*Z-*trn*M | IGS | A | NA | NA | 10 | 35812 | 10 | 35826 |
| **19** | *psb*Z-*trn*M | IGS | A | 10 | 35880 | 10 | 35862 | NA | NA |
| **20** | *ycf*3-*rps*4 | IGS | A | 10 | 44240 | 10 | 44222 | 10 | 44240 |
| **21** | *rps*4-*trn*T | IGS | T | 10 | 46317 | 11 | 46331 | 11 | 46327 |
| **22** | *trn*T-*ndh*J | IGS | T | 12 | 46685 | 12 | 46691 | 11 | 46687 |
| **23** | *trn*T-*ndh*J | IGS | A | 14 | 46727 | 12 | 46725 | 14 | 46728 |
| **24** | *ndh*C-*atp*E | IGS | T | 19 | 50513 | 17 | 50509 | 13 | 50515 |
| **25** | *ndh*C-*atp*E | IGS | T | NA | NA | 10 | 50639 | 10 | 50641 |
| **26** | *ndh*C-*atp*E | IGS | T | 10 | 51501 | 10 | 51496 | 15 | 51497 |
| **27** | *atp*B-*rbc*L | IGS | T | 11 | 54900 | 11 | 54904 | 11 | 54909 |
| **28** | *rbc*L-*acc*D | IGS | TTATTAG | NA | NA | 3 | 57019 | NA | NA |
| **29** | *acc*D-*psa*I | IGS | AAT | 4 | 59126 | 4 | 59137 | 4 | 59150 |
| **30** | *psa*I-*ycf*4 | IGS | T | 11 | 59516 | 11 | 59527 | 12 | 59540 |
| **31** | *pet*A-*psb*J | IGS | T | 17 | 63396 | 16 | 63407 | 14 | 63421 |
| **32** | *psb*E-*pet*L | IGS | A | 14 | 65641 | 15 | 65651 | 15 | 65663 |
| **33** | *rpl*33-*rps*18 | IGS | TA | 5 | 67984 | 5 | 67990 | 5 | 68008 |
| **34** | *rpl*33-*rps*18 | IGS | TATT | 3 | 68038 | 3 | 68044 | 3 | 68062 |
| **35** | *clp*P | Intron | T | 10 | 71605 | 11 | 71612 | 11 | 71630 |
| **36** | *pet*D-*rpo*A | IGS | A | 10 | 77974 | 10 | 78006 | 10 | 78000 |
| **37** | *rpo*A | CDS | T | 10 | 78215 | 10 | 78247 | 10 | 78241 |
| **38** | *rps*8-*rpl*14 | IGS | T | 12 | 80653 | 13 | 80685 | 17 | 80679 |
| **39** | *rpl*16 | Intron | TTTC | 3 | 82439 | 3 | 82472 | 3 | 82470 |
| **40** | *trn*L-*ndh*B | IGS | TCCTAA | 3 | 94113 | 3 | 94146 | 3 | 94144 |
| **41** | *ycf*15-*trn*N | IGS | A | 14 | 107686 | 14 | 107719 | 14 | 107723 |
| **42** | *ycf*1 | CDS | A | 10 | 109827 | 10 | 109860 | 10 | 109873 |
| **43** | *ycf*1 | CDS | A | 10 | 110281 | 10 | 110314 | 10 | 110327 |
| **44** | *ycf*1 | CDS | AATC | 3 | 112446 | 3 | 112479 | 3 | 112492 |
| **45** | *ndh*A | Intron | GATT | 3 | 116878 | 3 | 116911 | 3 | 116930 |
| **46** | *ndh*A | Intron | TTTC | 3 | 117284 | 3 | 117317 | 3 | 117336 |
| **47** | *trn*R-*ycf*15 | IGS | T | 14 | 129753 | 14 | 129810 | 14 | 129807 |
| **48** | *ndh*B-*ycf*2 | IGS | TTAGGA | 3 | 143322 | 3 | 143379 | 3 | 143382 |

CDS: coding sequence. IGS: intergenic region.
