# Supplementary material for: Phylogenetic relationships of Atractylodes lancea, A. chinensis and A. macrocephala, revealed by complete plastome and nuclear gene sequences
Source: PLoS One. 2020 Jan 28;15(1):e0227610. doi: 10.1371/journal.pone.0227610 (PMC6986703; doi:10.1371/journal.pone.0227610)
Supplement: S8 Table — (DOCX) [file pone.0227610.s008.docx]

**Table S8. Details of indels in the plastomes of the three *Atractylodes* species.**

| **No.** | **Position** | **Location** | **Region** | **Motif** | **Size (bp)** | **Direction^1^** | **Direction^2^** | **Direction^3^** |
| --- | --- | --- | --- | --- | --- | --- | --- | --- |
| **1** | 131 | *rpl*2-*trn*H | IGS | A | 1 | Insertion | Deletion | Deletion |
| **2** | 235 | *rpl*2-*trn*H | IGS | TTCATA | 6 | Insertion | Deletion | Deletion |
| **3** | 6966 | *rps*16-*trn*Q | IGS | AGGCTAAGCA | 10 | Deletion | Deletion | Insertion |
| **4** | 7917 | *psb*K-*psb*I | IGS | AATTG | 5 | Insertion | Deletion | Insertion |
| **5** | 10670 | *pet*N-*psb*M | IGS | ACTTTATAC/CCTTTATAC | 9 | Insertion | Deletion | Insertion |
| **6** | 12270 | *trn*E-*rpo*B | IGS | A | 1 | Deletion | Insertion | Deletion |
| **7** | 12492 | *trn*E-*rpo*B | IGS | GGCCATTTTAG | 11 | Insertion | Insertion | Deletion |
| **8** | 12937 | *trn*E-*rpo*B | IGS | T | 1 | Deletion | Deletion | Insertion |
| **9** | 17317 | *rpo*C1 | Intron | C | 1 | Deletion | Deletion | Insertion |
| **10** | 23525 | *rpo*C2-*rps*2 | IGS | T | 1 | Insertion | Insertion | Deletion |
| **11** | 25275 | *atp*I-*atp*H | IGS | CA | 2 | Insertion | Deletion | Insertion |
| **12** | 26345 | *atp*I-*atp*H | IGS | T | 1 | Insertion | Deletion | Deletion |
| **13** | 28285 | *atp*F-*atp*A | IGS | T | 1 | Insertion | Deletion | Deletion |
| **14** | 34983 | *psb*C-*trn*S | IGS | ACTCATTT | 8 | Deletion | Insertion | Insertion |
| **15** | 35831 | *psb*Z-*trn*M | IGS | A | 1 | Deletion | Insertion | Insertion |
| **16** | 35880 | *psb*Z-*trn*M | IGS | A | 1 | Insertion | Insertion | Deletion |
| **17** | 36116 | *psb*Z-*trn*M | IGS | AAAAT | 5 | Deletion | Deletion | Insertion |
| **18** | 46176 | *rps*4-*trn*T | IGS | TATTAAAGAATAATGAGATGAG | 22 | Deletion | Insertion | Deletion |
| **19** | 46294 | *rps*4-*trn*T | IGS | ATAAATCAAT | 10 | Deletion | Insertion | Insertion |
| **20** | 46322 | *rps*4-*trn*T | IGS | T | 1 | Deletion | Insertion | Insertion |
| **21** | 46672 | *trn*T-*ndh*J | IGS | GAAATATTC | 9 | Insertion | Deletion | Deletion |
| **22** | 46685 | *trn*T-*ndh*J | IGS | T | 1 | Insertion | Insertion | Deletion |
| **23** | 46699 | *trn*T-*ndh*J | IGS | TTTTATTT | 8 | Insertion | Deletion | Insertion |
| **24** | 46727 | *trn*T-*ndh*J | IGS | AA | 2 | Insertion | Deletion | Insertion |
| **25** | 48549 | *trn*T-*ndh*J | IGS | T | 1 | Deletion | Deletion | Insertion |
| **26** | 50645 | *ndh*C-*atp*E | IGS | T | 1 | Deletion | Insertion | Insertion |
| **27** | 51497 | *ndh*C-*atp*E | IGS | TTTA | 4 | Deletion | Deletion | Insertion |
| **28** | 54971 | *atp*B-rbcL | IGS | ACAACATATATCACT | 16 | Deletion | Deletion | Insertion |
| **29** | 57024 | rbcL-*acc*D | IGS | TTATTAG | 7 | Deletion | Insertion | Deletion |
| **30** | 57051 | rbcL-*acc*D | IGS | A | 1 | Insertion | Insertion | Deletion |
| **31** | 59525 | *psa*I-*ycf*4 | IGS | T | 1 | Deletion | Deletion | Insertion |
| **32** | 65650 | *psb*E-*pet*L | IGS | A | 1 | Deletion | Insertion | Insertion |
| **33** | 67960 | *rpl*33-*rps*18 | IGS | TCTTAC | 6 | Deletion | Deletion | Insertion |
| **34** | 67986 | *rpl*33-*rps*18 | IGS | ATTAT | 5 | Insertion | Deletion | Deletion |
| **35** | 71271 | *clp*P | Intron | A | 1 | Deletion | Insertion | Insertion |
| **36** | 71620 | *clp*P | Intron | T | 1 | Deletion | Insertion | Insertion |
| **37** | 74426 | *psb*N | CDS | TTCAATTAGTCCCCATGTTCCTCG | 24 | Deletion | Insertion | Deletion |
| **38** | 82783 | *rpl*16-*rps*3 | IGS | T | 1 | Deletion | Deletion | Insertion |
| **39** | 84025 | *rpl*22-*rps*19 | IGS | C | 1 | Insertion | Insertion | Deletion |
| **40** | 98494 | *pet*A-*psb*J | IGS | TTATAA | 6 | Deletion | Deletion | Insertion |
| **41** | 109632 | *ycf*1 | CDS | GAAGAAGAC | 9 | Deletion | Deletion | Insertion |
| **42** | 113271 | *ycf*1 | CDS | TTTGAA | 6 | Deletion | Deletion | Insertion |
| **43** | 128040 | *ndh*F | CDS | TTTTATTAATTTATTACTTTTTG | 23 | Deletion | Insertion | Deletion |
| **44** | 138982 | *ycf*15-*rps*12 | IGS | ATTATA | 6 | Deletion | Deletion | Insertion |

CDS: coding sequence. IGS: intergenic region.

^1^: *A. lancea*, ^2^: *A. chinensis*, ^3^: *A. macrocephala*
