# Supplementary material for: Phylogenetic relationships of Atractylodes lancea, A. chinensis and A. macrocephala, revealed by complete plastome and nuclear gene sequences
Source: PLoS One. 2020 Jan 28;15(1):e0227610. doi: 10.1371/journal.pone.0227610 (PMC6986703; doi:10.1371/journal.pone.0227610)
Supplement: S9 Table — (DOCX) [file pone.0227610.s009.docx]

**Table S9. Variation sites found among the plastomes of the three *Atractylodes* species.**

| **Number** | ***A. lancea*** | | ***A. chinensis*** | | ***A. macrocephala*** | |
| --- | --- | --- | --- | --- | --- | --- |
|  | **Position** | **Nucleotide** | **Position** | **Nucleotide** | **Position** | **Nucleotide** |
| **1** | T | 193 | T | 192 | A | 192 |
| **2** | T | 444 | C | 437 | T | 437 |
| **3** | A | 1699 | A | 1692 | C | 1692 |
| **4** | T | 1986 | T | 1979 | A | 1979 |
| **5** | A | 2164 | A | 2157 | G | 2157 |
| **6** | A | 2940 | G | 2933 | G | 2933 |
| **7** | A | 3336 | C | 3329 | A | 3329 |
| **8** | A | 3864 | A | 3857 | T | 3857 |
| **9** | C | 4068 | A | 4061 | C | 4061 |
| **10** | C | 4307 | C | 4300 | A | 4300 |
| **11** | T | 6432 | G | 6425 | T | 6422 |
| **12** | A | 8353 | A | 8340 | G | 8353 |
| **13** | C | 9597 | A | 9584 | A | 9597 |
| **14** | A | 10035 | C | 10022 | C | 10035 |
| **15** | C | 11482 | A | 11460 | C | 11482 |
| **16** | A | 11552 | A | 11530 | C | 11552 |
| **17** | A | 12052 | A | 12030 | G | 12052 |
| **18** | A | 12642 | G | 12621 | G | 12631 |
| **19** | T | 13312 | G | 13291 | T | 13302 |
| **20** | A | 13673 | C | 13652 | C | 13663 |
| **21** | A | 13879 | A | 13858 | C | 13869 |
| **22** | A | 14612 | A | 14591 | C | 14602 |
| **23** | T | 15032 | G | 15011 | G | 15022 |
| **24** | A | 16450 | A | 16429 | G | 16440 |
| **25** | T | 17323 | G | 17302 | G | 17314 |
| **26** | G | 19063 | G | 19042 | A | 19054 |
| **27** | A | 20245 | C | 20224 | A | 20236 |
| **28** | C | 20521 | C | 20500 | T | 20512 |
| **29** | T | 25754 | T | 25731 | C | 25744 |
| **30** | G | 26900 | A | 26876 | A | 26889 |
| **31** | G | 30252 | G | 30225 | T | 30239 |
| **32** | A | 30688 | A | 30661 | G | 30675 |
| **33** | C | 30885 | A | 30858 | A | 30872 |
| **34** | A | 31178 | A | 31151 | G | 31165 |
| **35** | T | 31400 | T | 31373 | C | 31387 |
| **36** | A | 31590 | A | 31563 | C | 31577 |
| **37** | A | 33877 | G | 33850 | G | 33864 |
| **38** | A | 34981 | A | 34954 | T | 34968 |
| **39** | C | 35071 | T | 35052 | T | 35066 |
| **40** | C | 41758 | A | 41740 | A | 41758 |
| **41** | A | 41964 | A | 41946 | G | 41964 |
| **42** | C | 42148 | C | 42130 | A | 42148 |
| **43** | A | 42917 | A | 42899 | C | 42917 |
| **44** | C | 45013 | C | 44995 | T | 45013 |
| **45** | A | 45589 | A | 45571 | G | 45589 |
| **46** | A | 47196 | G | 47192 | A | 47197 |
| **47** | T | 47197 | G | 47193 | G | 47198 |
| **48** | A | 47524 | C | 47520 | A | 47525 |
| **49** | T | 47569 | T | 47565 | C | 47570 |
| **50** | G | 47683 | G | 47679 | A | 47684 |
| **51** | A | 51499 | A | 51494 | T | 51500 |
| **52** | G | 51992 | G | 51987 | C | 51993 |
| **53** | T | 53752 | C | 53747 | C | 53753 |
| **54** | A | 54530 | T | 54525 | T | 54531 |
| **55** | G | 54531 | A | 54526 | A | 54532 |
| **56** | A | 54615 | G | 54619 | G | 54624 |
| **57** | T | 54936 | T | 54940 | G | 54945 |
| **58** | A | 55544 | A | 55548 | C | 55569 |
| **59** | A | 57810 | A | 57821 | G | 57834 |
| **60** | C | 58510 | C | 58521 | T | 58534 |
| **61** | T | 60044 | T | 60055 | C | 60069 |
| **62** | G | 60960 | T | 60971 | G | 60985 |
| **63** | T | 62812 | C | 62823 | C | 62837 |
| **64** | G | 63521 | G | 63531 | A | 63543 |
| **65** | C | 63840 | A | 63850 | A | 63862 |
| **66** | G | 65810 | G | 65821 | A | 65833 |
| **67** | T | 67541 | G | 67552 | G | 67564 |
| **68** | T | 67940 | C | 67951 | T | 67963 |
| **69** | G | 67965 | T | 67976 | G | 67994 |
| **70** | A | 68063 | A | 68069 | G | 68087 |
| **71** | C | 68502 | C | 68508 | A | 68526 |
| **72** | T | 69195 | G | 69201 | G | 69219 |
| **73** | G | 74081 | G | 74089 | T | 74107 |
| **74** | C | 74988 | C | 75020 | A | 75014 |
| **75** | T | 75502 | G | 75534 | G | 75528 |
| **76** | C | 75693 | C | 75725 | T | 75719 |
| **77** | T | 78380 | G | 78412 | T | 78406 |
| **78** | G | 78665 | G | 78697 | C | 78691 |
| **79** | C | 78773 | C | 78805 | T | 78799 |
| **80** | C | 82024 | C | 81920 | T | 82055 |
| **81** | G | 83683 | G | 83578 | T | 83715 |
| **82** | G | 107248 | A | 107137 | A | 107285 |
| **83** | A | 107670 | T | 107559 | T | 107707 |
| **84** | T | 108110 | G | 107999 | G | 108147 |
| **85** | A | 109194 | A | 109083 | G | 109231 |
| **86** | G | 109459 | G | 109348 | A | 109496 |
| **87** | T | 109497 | T | 109386 | A | 109534 |
| **88** | G | 110231 | A | 110111 | G | 110277 |
| **89** | G | 110258 | T | 110138 | G | 110304 |
| **90** | T | 110477 | A | 110357 | T | 110523 |
| **91** | T | 110854 | T | 110734 | C | 110900 |
| **92** | A | 111570 | G | 111450 | G | 111616 |
| **93** | A | 113181 | G | 113061 | G | 113227 |
| **94** | A | 115250 | A | 115124 | G | 115302 |
| **95** | T | 115807 | C | 115681 | C | 115859 |
| **96** | G | 118036 | G | 117910 | A | 118088 |
| **97** | A | 118566 | A | 118440 | G | 118618 |
| **98** | C | 118619 | C | 118493 | T | 118671 |
| **99** | G | 121149 | A | 121023 | G | 121201 |
| **100** | A | 121805 | G | 121679 | G | 121857 |
| **101** | A | 122946 | T | 122820 | A | 122998 |
| **102** | A | 123599 | A | 123473 | C | 123651 |
| **103** | A | 123848 | C | 123722 | C | 123900 |
| **104** | T | 124224 | T | 124098 | G | 124276 |
| **105** | T | 124974 | T | 124848 | C | 125026 |
| **106** | G | 125224 | G | 125098 | T | 125276 |
| **107** | C | 125541 | T | 125415 | C | 125595 |
| **108** | T | 128256 | T | 128129 | C | 128310 |
| **109** | A | 129340 | C | 129213 | C | 129394 |
| **110** | T | 129780 | A | 129653 | A | 129834 |
| **111** | C | 130202 | T | 130075 | T | 130256 |
