# Supplementary material for: Phylogenetic relationships of Atractylodes lancea, A. chinensis and A. macrocephala, revealed by complete plastome and nuclear gene sequences
Source: PLoS One. 2020 Jan 28;15(1):e0227610. doi: 10.1371/journal.pone.0227610 (PMC6986703; doi:10.1371/journal.pone.0227610)

**Fig S1. Codon contents of 20 amino acid and stop codons in all protein-coding genes of the chloroplast genomes of the three *Atractylodes* species.** The x-axis shows the amino acids and their codons. The y-axis shows the RSCU values. The columns represent the amino acids of *A. lancea*, *A. chinensis*, and *A. macrocephala* (from left to right). Different codons are labeled using different colors.


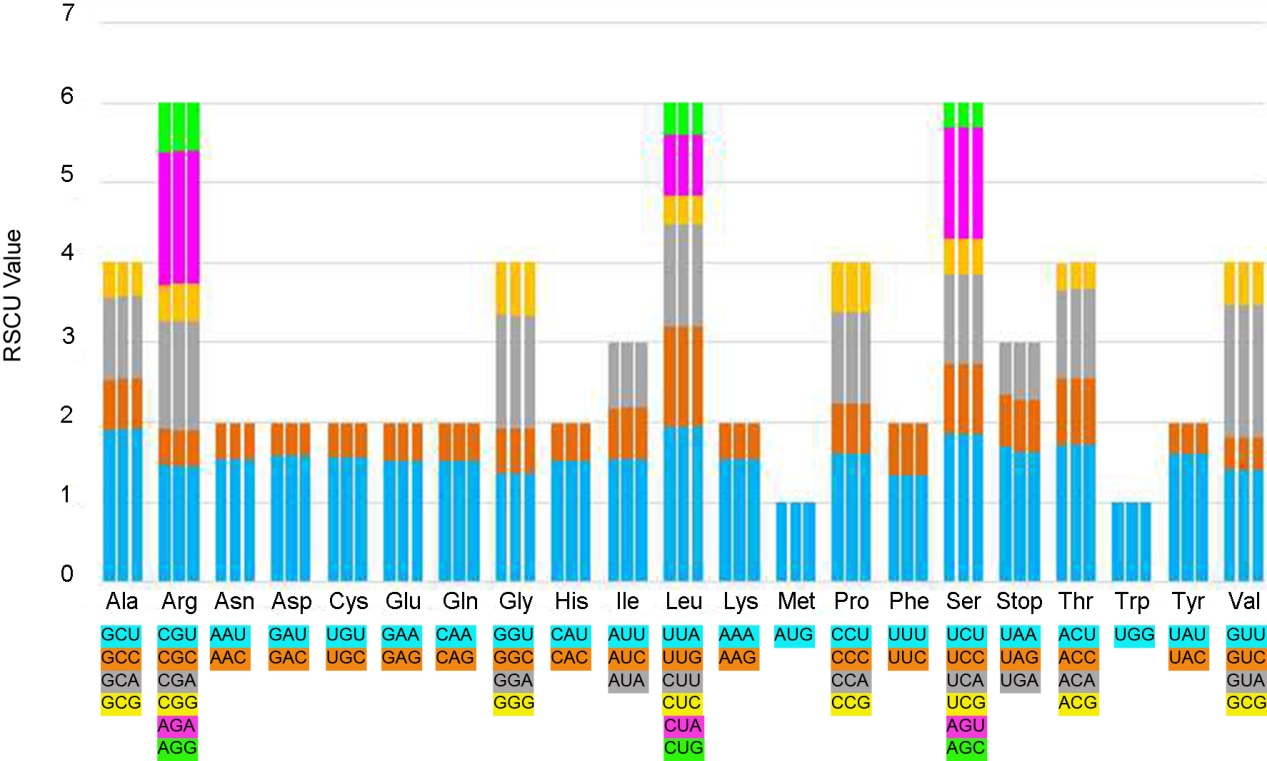

Supplement: S1 Fig — The x-axis shows the amino acids and their codons. The y-axis shows the RSCU values. The columns represent the amino acids of A. lancea, A. chinensis, and A. macrocephala (from left to right). Different codons are labeled using different colors. (DOCX) [file pone.0227610.s010.docx]
