# Supplementary material for: Phylogenetic relationships of Atractylodes lancea, A. chinensis and A. macrocephala, revealed by complete plastome and nuclear gene sequences
Source: PLoS One. 2020 Jan 28;15(1):e0227610. doi: 10.1371/journal.pone.0227610 (PMC6986703; doi:10.1371/journal.pone.0227610)

**Fig S2. Lengths and number of repeat sequences found in the three chloroplast genomes.** REPuter was used to identify the repeat sequences with length ≥ 30 bp and sequence identity ≥ 90 % in the chloroplast genomes. The repeats were further binned according to their length. The x-axis shows the species, the type of repeat (F or P), and the bin of the repeats based on length. The y-axis shows the number of repeats in each bin. The numbers of repeats in each bin are also shown on the top of the corresponding columns. F: forward repeat; P: palindrome repeat; Green: repeat length in the range of 30–39; Red: repeat length in the range of 40–49; Yellow: repeat length in the range of 50–59.


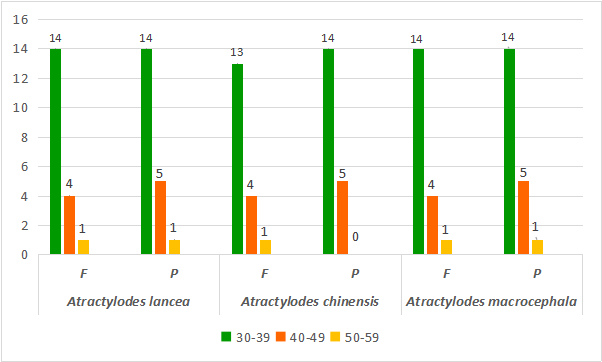

Supplement: S2 Fig — REPuter was used to identify the repeat sequences with length ≥ 30 bp and sequence identity ≥ 90% in the plastomes. The repeats were further binned according to their length. The x-axis shows the species, the type of repeat (F or P), and the bin of the repeats based on length. The y-axis shows the number of repeats in each bin. The numbers of repeats in each bin are also shown on the top of the corresponding columns. F: forward repeat; P: palindrome repeat; Green: repeat length in the range of 30–39; Red: repeat length in the range of 40–49; Yellow: repeat length in the range of 50–59. (DOCX) [file pone.0227610.s011.docx]
