# Supplementary material for: Phylogenetic relationships of Atractylodes lancea, A. chinensis and A. macrocephala, revealed by complete plastome and nuclear gene sequences
Source: PLoS One. 2020 Jan 28;15(1):e0227610. doi: 10.1371/journal.pone.0227610 (PMC6986703; doi:10.1371/journal.pone.0227610)
Supplement: S3 Fig — AL_AK: A. lancea collected from Ankang City, Shanxi Province. AL_SZ: A. lancea collected from Suizhou City, Hubei Province. AL_HB: A. lancea collected from Harbin City, Heilongjiang Province. AL_BZ: A. lancea collected from Bozhou City, Anhui Province. AM_BZ: A. macrocephala collected from Bozhou City, Anhui Province. AM_ES: A. macrocephala collected from Enshi City, Hubei Provincce. AM_LH: A. macrocephala collected from Luohe City, Henan Province. AC_LY: A. chinensis collected from Luoyang City, Henan Province. AM_BZ: A. macrocephala collected from Bozhou City, Anhui Province. AM_ES: A. macrocephala collected from Enshi City, Hubei Provincce. AM_LH: A. macrocephala collected from Luohe City, Henan Province. AC_LY: A. chinensis collected from Luoyang City, Henan Province. AC_FS: A. chinensis collected from Fushun City, Liaoning Province. AC_CF: A. chinensis collected from Chifeng City, Inner Mongolia. (DOCX) [file pone.0227610.s012.docx]

**Fig S3. Validation of the marker cz11 using samples from different individual plant of the three *Atractylodes* species collected from different origins.** AL_AK: *A. lancea* collected from Ankang City, Shanxi Province. AL_SZ: *A. lancea* collected from Suizhou City, Hubei Province. AL_HB: *A. lancea* collected from Harbin City, Heilongjiang Province. AL_BZ: *A. lancea* collected from Bozhou City, Anhui Province. AM_BZ: *A. macrocephala* collected from Bozhou City, Anhui Province. AM_ES: *A. macrocephala* collected from Enshi City, Hubei Provincce. AM_LH: *A. macrocephala* collected from Luohe City, Henan Province. AC_LY: *A. chinensis* collected from Luoyang City, Henan Province. AM_BZ: *A. macrocephala* collected from Bozhou City, Anhui Province. AM_ES: A. macrocephala collected from Enshi City, Hubei Provincce. AM_LH: A. macrocephala collected from Luohe City, Henan Province. AC_LY: *A. chinensis* collected from Luoyang City, Henan Province. AC_FS: *A. chinensis* collected from Fushun City, Liaoning Province. AC_CF: *A. chinensis* collected from Chifeng City, Inner Mongolia.

AL_AK1 : TCCGCCCTTATCTTAC------AAGGAAAGGGAAAAAATGACATTATAT-----ATATAACATATTTAACATAGTTAAAGATAATTATAAACAAACCAAATCCTATTTATTTATTCTAATTAGAGATACAGCTGAAATAGGATTTTAGGGAG
AL_AK2 : CCCGCCCTTATCTTAC------AAGGAAAGGGAAAAAATGACATTATAT-----ATATAACATATTTAACATAGTTAAAGATAATTATAAACAAACCAAATCCTATTTATTTATTCTAATTAGAGATACAGCTGAAATAGGATTTTAGGGAG
AL_AK3 : TCCGCCCTTATCTTAC------AAGGAAAGGGAAAAAATGACATTATAT-----ATATAACATATTTAACATAGTTAAAGATAATTATAAACAAACCAAATCCTATTTATTTATTCTAATTAGAGATACGGCTGAAATAGGATTTTAGGGAG
AL_AK4 : TCCGCCCTTATCTTAC------AAGGAAAGGGAAAAAATGACATTATATTATATATATAACATATTTAACATAGTTAAAGATAATTATAAACAAACCAAATCCTATTTATTTATTCTAATTAGAGATACAGCTGAAATAGGATTTTAGGGAG
AL_AK5 : TCCGCCCTTATCTTAC------AAGGAAAGGGAAAAAATGACATTATAT-----ATATAACATATTTAACATAGTTAAAGATAATTATAAACAAACCAAATCCTATTTATTTATTCTAATTAGAGATACGGCTGAAATAGGATTTTAGGGAG
AL_AK6 : TCCGCCCTTATCTTAC------AAGGAAAGGGAAAAAATGACATTATAT-----ATATAACATATTTAACATAGTTAAAGATAATTATAAACAAACCAAATCCTATTTATTTATTCTAATTAGAGATACAGCTGAAATAGGATTTTAGGGAG
AL_AK7 : TCCGCCCTTATCTTAC------AAGGAAAGGGAAAAAATGACATTATAT-----ATATAACATATTTAACATAGTTAAAGATAATTATAAACAAACCAAATCCTATTTATTTATTCTAATTAGAGATACGGCTGAAATAGGATTTTAGGGAG
AL_AK8 : TCCGCCCTTATCTTAC------AAGGAAAGGGAAAAAATGACATTATAT-----ATATAACATATTTAACATAGTTAAAGATAATTATAAACAAACCAAATCCTATTTATTTATTCTAATTAGAGATACAGCTGAAATAGGATTTTAGGGAG
AL_AK9 : TCCGCCCTTATCTTAC------AAGGAAAGGGAAAAAATGACATTATAT-----ATATAACATATTTAACATAGTTAAAGATAATTATAAACAAACCAAATCCTATTTATTTATTCTAATTAGAGATACGGCTGAAATAGGATTTTAGGGAG
AL_AK10 : TCCGCCCTTATCTTAC------AAGGAAAGGGAAAAAATGACATTATATTATATATATAACATATTTAACATAGTTAAAGATAATTATAAACAAACCAAATCCTATTTATTTATTCTAATTAGAGATACAGCTGAAATAGGATTTTAGGGAG
AL_AK11 : TCCGCCCTTATCTTAC------AAGGAAAGGGAAAAAATGACATTATAT-----ATATAACATATTTAACATAGTTAAAGATAATTATAAACAAACCAAATCCTATTTATTTATTCTAATTAGAGATACAGCTGAAATAGGATTTTAGGGAG
AL_AK12 : TCCGCCCTTATCTTAC------AAGGAAAGGGAAAAAATGACATTATAT-----ATATAACATATTTAACATAGTTAAAGATAATTATAAACAAACCAAATCCTATTTATTTATTCTAATTAGAGATACGGCTGAAATAGGATTTTAGGGAG
AL_AK13 : TCCGCCCTTATCTTAC------AAGGAAAGGGAAAAAATGACATTATAT-----ATATAACATATTTAACATAGTTAAAGATAATTATAAACAAACCAAATCCTATTTATTTATTCTAATTAGAGATACGGCTGAAATAGGATTTTAGGGAG
AL_AK14 : TCCGCCCTTATCTTAC------AAGGAAAGGGAAAAAATGACATTATAT-----ATATAACATATTTAACATAGTTAAAGATAATTATAAACAAACCAAATCCTATTTATTTATTCTAATTAGAGATACGGCTGAAATAGGATTTTAGGGAG
AL_AK15 : TCCGCCCTTATCTTAC------AAGGAAAGGGAAAAAATGACATTATAT-----ATATAACATATTTAACATAGTTAAAGATAATTATAAACAAACCAAATCCTATTTATTTATTCTAATTAGAGATACGGCTGAAATAGGATTTTAGGGAG
AL_AK16 : TCCGCCCTTATCTTAC------AAGGAAAGGGAAAAAATGACATTATAT-----ATATAACATATTTAACATAGTTAAAGATAATTATAAACAAACCAAATCCTATTTATTTATTCTAATTAGAGATACGGCTGAAATAGGATTTTAGGGAG
AL_AK17 : TCCGCCCTTATCTTAC------AAGGAAAGGGAAAAAATGACATTATAT-----ATATAACATATTTAACATAGTTAAAGATAATTATAAACAAACCAAATCCTATTTATTTATTCTAATTAGAGATACGGCTGAAATAGGATTTTAGGGAG
AL_AK18 : TCCGCCCTTATCTTAC------AAGGAAAGGGAAAAAATGACATTATAT-----ATATAACATATTTAACATAGTTAAAGATAATTATAAACAAACCAAATCCTATTTATTTATTCTAATTAGAGATACGGCTGAAATAGGATTTTAGGGAG
AL_HB1 : TCCGCCCTTATCTTAC------AAGGAAAGGGAAAAAATGACATTATATTATATATATAACATATTTAACATAGTTAAAGATAATTATAAACAAAGCAAATCCTATTTATTTATTCTAATTAGAGATACAGCTGAAATAGGATTTTAGGGAG
AL_HB2 : TCCGCCCTTATCTTAC------AAGGAAAGGGAAAAAATGACATTATATTATATATATAACATATTTAACATAGTTAAAGATAATTATAAACAAAGCAAATCCTATTTATTTATTCTAATTAGAGATACAGCTGAAATAGGATTTTAGGGAG
AL_HB3 : TCCGCCCTTATCTTAC------AAGGAAAGGGAAAAAATGACATTATAT-----ATATAACATATTTAACATAGTTAAAGATAATTATAAACAAACCAAATCCTATTTATTTATTCTAATTAGAGATACGGCTGAAATAGGATTTTAGGGAG
AL_HB4 : CCCGCCCTTATCTTAC------AAGGAAAGGGAAAAAATGACATTATAT-----ATATAACATATTTAACATAGTTAAAGATAATTATAAACAAACCAAATCCTATTTATTTATTCTAATTAGAGATACAGCTGAAATAGGATTTTAGGGAG
AL_HB5 : CCCGCCCTTATCTTAC------AAGGAAAGGGAAAAAATGACATTATAT-----ATATAACATATTTAACATAGTTAAAGATAATTATAAACAAACCAAATCCTATTTATTTATTCTAATTAGAGATACAGCTGAAATAGGATTTTAGGGAG
AL_HB6 : TCCGCCCTTATCTTAC------AAGGAAAGGGAAAAAATGACATTATAT-----ATATAACATATTTAACATAGTTAAAGATAATTATAAACAAACCAAATCCTATTTATTTATTCTAATTAGAGATACGGCTGAAATAGGATTTTAGGGAG
AL_HB7 : TCCGCCCTTATCTTAC------AAGGAAAGGGAAAAAATGACATTATAT-----ATATAACATATTTAACATAGTTAAAGATAATTATAAACAAACCAAATCCTATTTATTTATTCTAATTAGAGATACGGCTGAAATAGGATTTTAGGGAG
AL_BZ1 : TCCGCCCTTATCTTAC------AAGGAAAGGGAAAAAATGACATTATAT-----ATATAACATATTTAACATAGTTAAAGATAATTATAAACAAACCAAATCCTATTTATTTATTCTAATTAGAGATACGGCTGAAATAGGATTTTAGGGAG
AL_BZ2 : TCCGCCCTTATCTTAC------AAGGAAAGGGAAAAAATGACATTATAT-----ATATAACATATTTAACATAGTTAAAGATAATTATAAACAAACCAAATCCTATTTATTTATTCTAATTAGAGATACGGCTGAAATAGGATTTTAGGGAG
AL_BZ3 : TCCGCCCTTATCTTAC------AAGGAAAGGGAAAAAATGACATTATAT-----ATATAACATATTTAACATAGTTAAAGATAATTATAAACAAACCAAATCCTATTTATTTATTCTAATTAGAGATACGGCTGAAATAGGATTTTAGGGAG
AL_BZ4 : TCCGCCCTTATCTTAC------AAGGAAAGGGAAAAAATGACATTATAT-----ATATAACATATTTAACATAGTTAAAGATAATTATAAACAAACCAAATCCTATTTATTTATTCTAATTAGAGATACGGCTGAAATAGGATTTTAGGGAG
AL_BZ5 : TCCGCCCTTATCTTAC------AAGGAAAGGGAAAAAATGACATTATAT-----ATATAACATATTTAACATAGTTAAAGATAATTATAAACAAACCAAATCCTATTTATTTATTCTAATTAGAGATACGGCTGAAATAGGATTTTAGGGAG
AL_BZ6 : TCCGCCCTTATCTTAC------AAGGAAAGGGAAAAAATGACATTATAT-----ATATAACATATTTAACATAGTTAAAGATAATTATAAACAAACCAAATCCTATTTATTTATTCTAATTAGAGATACGGCTGAAATAGGATTTTAGGGAG
AL_BZ7 : TCCGCCCTTATCTTAC------AAGGAAAGGGAAAAAATGACATTATAT-----ATATAACATATTTAACATAGTTAAAGATAATTATAAACAAACCAAATCCTATTTATTTATTCTAATTAGAGATACGGCTGAAATAGGATTTTAGGGAG
AL_BZ8 : TCCGCCCTTATCTTAC------AAGGAAAGGGAAAAAATGACATTATAT-----ATATAACATATTTAACATAGTTAAAGATAATTATAAACAAACCAAATCCTATTTATTTATTCTAATTAGAGATACGGCTGAAATAGGATTTTAGGGAG
AL_BZ9 : TCCGCCCTTATCTTAC------AAGGAAAGGGAAAAAATGACATTATAT-----ATATAACATATTTAACATAGTTAAAGATAATTATAAACAAACCAAATCCTATTTATTTATTCTAATTAGAGATACGGCTGAAATAGGATTTTAGGGAG
AL_SZ1 : TCCGCCCTTATCTTAC------AAGGAAAGGGAAAAAATGACATTATAT-----ATATAACATATTTAACATAGTTAAAGATAATTATAAACAAACCAAATCCTATTTATTTATTCTAATTAGAGATACGGCTGAAATAGGATTTTAGGGAG
AL_SZ2 : TCCGCCCTTATCTTAC------AAGGAAAGGGAAAAAATGACATTATAT-----ATATAACATATTTAACATAGTTAAAGATAATTATAAACAAACCAAATCCTATTTATTTATTCTAATTAGAGATACGGCTGAAATAGGATTTTAGGGAG
AL_SZ3 : TCCGCCCTTATCTTAC------AAGGAAAGGGAAAAAATGACATTATAT-----ATATAACATATTTAACATAGTTAAAGATAATTATAAACAAACCAAATCCTATTTATTTATTCTAATTAGAGATACGTCTGAAATAGGATTTTAGGGAG
AL_SZ4 : TCCGCCCTTATCTTAC------AAGGAAAGGGAAAAAATGACATTATAT-----ATATAACATATTTAACATAGTTAAAGATAATTATAAACAAACCAAATCCTATTTATTTATTCTAATTAGAGATACGGCTGAAATAGGATTTTAGGGAG
AL_SZ5 : TCCGCCCTTATCTTAC------AAGGAAAGGGAAAAAATGACATTATAT-----ATATAACATATTTAACATAGTTAAAGATAATTATAAACAAACCAAATCCTATTTATTTATTCTAATTAGAGATACGGCTGAAATAGGATTTTAGGGAG
AL_SZ6 : TCCGCCCTTATCTTAC------AAGGAAAGGGAAAAAATGACATTATAT-----ATATAACATATTTAACATAGTTAAAGATAATTATAAACAAACCAAATCCTATTTATTTATTCTAATTAGAGATACAGCTGAAATAGGATTTTAGGGAG
AM_BZ1 : TCCGCCCTTATCTTACTCTTACAAGGAAAGGGAAAAAATGACATTATAT-----ATATAACATATTTAACATAGTTAAAGATAATTATAAACAAACCAAATCCTATTTATTTATTCTAATTAGAGATACGGCTGAAATAGGATTTTAGGGAG
AM_BZ2 : TCCGCCCTTATCTTACTCTTACAAGGAAAGGGAAAAAATGACATTATAT-----ATATAACATATTTAACATAGTTAAAGATAATTATAAACAAACCAAATCCTATTTATTTATTCTAATTAGAGATACGGCTGAAATAGGATTTTAGGGAG
AM_BZ3 : TCCGCCCTTATCTTACTCTTACAAGGAAAGGGAAAAAATGACATTATAT-----ATATAACATATTTAACATAGTTAAAGATAATTATAAACAAACCAAATCCTATTTATTTATTCTAATTAGAGATACGGCTGAAATAGGATTTTAGGGAG
AM_BZ4 : TCCGCCCTTATCTTACTCTTACAAGGAAAGGGAAAAAATGACATTATAT-----ATATAACATATTTAACATAGTTAAAGATAATTATAAACAAACCAAATCCTATTTATTTATTCTAATTAGAGATACGGCTGAAATAGGATTTTAGGGAG
AM_BZ5 : TCCGCCCTTATCTTACTCTTACAAGGAAAGGGAAAAAATGACATTATAT-----ATATAACATATTTAACATAGTTAAAGATAATTATAAACAAACCAAATCCTATTTATTTATTCTAATTAGAGATACGGCTGAAATAGGATTTTAGGGAG
AM_BZ6 : TCCGCCCTTATCTTACTCTTACAAGGAAAGGGAAAAAATGACATTATAT-----ATATAACATATTTAACATAGTTAAAGATAATTATAAACAAACCAAATCCTATTTATTTATTCTAATTAGAGATACGGCTGAAATAGGATTTTAGGGAG
AM_ES1 : TCCGCCCTTATCTTACTCTTACAAGGAAAGGGAAAAAATGACATTATAT-----ATATAACATATTTAACATAGTTAAAGATAATTATAAACAAACCAAATCCTATTTATTTATTCTAATTAGAGATACGGCTGAAATAGGATTTTAGGGAG
AM_ES2 : TCCGCCCTTATCTTACTCTTACAAGGAAAGGGAAAAAATGACATTATAT-----ATATAACATATTTAACATAGTTAAAGATAATTATAAACAAACCAAATCCTATTTATTTATTCTAATTAGAGATACGGCTGAAATAGGATTTTAGGGAG
AM_ES3 : TCCGCCCTTATCTTACTCTTACAAGGAAAGGGAAAAAATGACATTATAT-----ATATAACATATTTAACATAGTTAAAGATAATTATAAACAAACCAAATCCTATTTATTTATTCTAATTAGAGATACGGCTGAAATAGGATTTTAGGGAG
AM_ES4 : TCCGCCCTTATCTTACTCTTACAAGGAAAGGGAAAAAATGACATTATAT-----ATATAACATATTTAACATAGTTAAAGATAATTATAAACAAACCAAATCCTATTTATTTATTCTAATTAGAGATACGGCTGAAATAGGATTTTAGGGAG
AM_ES5 : TCCGCCCTTATCTTACTCTTACAAGGAAAGGGAAAAAATGACATTATAT-----ATATAACATATTTAACATAGTTAAAGATAATTATAAACAAACCAAATCCTATTTATTTATTCTAATTAGAGATACGGCTGAAATAGGATTTTAGGGAG
AM_ES6 : TCCGCCCTTATCTTACTCTTACAAGGAAAGGGAAAAAATGACATTATAT-----ATATAACATATTTAACATAGTTAAAGATAATTATAAACAAACCAAATCCTATTTATTTATTCTAATTAGAGATACGGCTGAAATAGGATTTTAGGGAG
AM_LH1 : TCCGCCCTTATCTTACTCTTACAAGGAAAGGGAAAAAATGACATTATAT-----ATATAACATATTTAACATAGTTAAAGATAATTATAAACAAACCAAATCCTATTTATTTATTCTAATTAGAGATACGGCTGAAATAGGATTTTAGGGAG
AM_LH2 : TCCGCCCTTATCTTACTCTTACAAGGAAAGGGAAAAAATGACATTATAT-----ATATAACATATTTAACATAGTTAAAGATAATTATAAACAAACCAAATCCTATTTATTTATTCTAATTAGAGATACGGCTGAAATAGGATTTTAGGGAG
AM_LH3 : TCCGCCCTTATCTTACTCTTACAAGGAAAGGGAAAAAATGACATTATAT-----ATATAACATATTTAACATAGTTAAAGATAATTATAAACAAACCAAATCCTATTTATTTATTCTAATTAGAGATACGGCTGAAATAGGATTTTAGGGAG
AM_LH4 : TCCGCCCTTATCTTACTCTTACAAGGAAAGGGAAAAAATGACATTATAT-----ATATAACATATTTAACATAGTTAAAGATAATTATAAACAAACCAAATCCTATTTATTTATTCTAATTAGAGATACGGCTGAAATAGGATTTTAGGGAG
AM_LH5 : TCCGCCCTTATCTTACTCTTACAAGGAAAGGGAAAAAATGACATTATAT-----ATATAACATATTTAACATAGTTAAAGATAATTATAAACAAACCAAATCCTATTTATTTATTCTAATTAGAGATACGGCTGAAATAGGATTTTAGGGAG
AM_LH6 : TCCGCCCTTATCTTACTCTTACAAGGAAAGGGAAAAAATGACATTATAT-----ATATAACATATTTAACATAGTTAAAGATAATTATAAACAAACCAAATCCTATTTATTTATTCTAATTAGAGATACGGCTGAAATAGGATTTTAGGGAG
AC_LY1 : TCCGCCCTTATCTTAC------AAGGAAAGGGAAAAAATGACATTATAT-----ATATAACATATTTAACATAGTTAAAGATAATTATAAACAAACCAAATCCTATTTATTTATTCTAATTAGAGATACGGCTGAAATAGGATTTTAGGGAG
AC_LY2 : TCCGCCCTTATCTTAC------AAGGAAAGGGAAAAAATGACATTATAT-----ATATAACATATTTAACATAGTTAAAGATAATTATAAACAAACCAAATCCTATTTATTTATTCTAATTAGAGATACGGCTGAAATAGGATTTTAGGGAG
AC_LY3 : TCCGCCCTTATCTTAC------AAGGAAAGGGAAAAAATGACATTATAT-----ATATAACATATTTAACATAGTTAAAGATAATTATAAACAAACCAAATCCTATTTATTTATTCTAATTAGAGATACGGCTGAAATAGGATTTTAGGGAG
AC_LY4 : TCCGCCCTTATCTTAC------AAGGAAAGGGAAAAAATGACATTATAT-----ATATAACATATTTAACATAGTTAAAGATAATTATAAACAAACCAAATCCTATTTATTTATTCTAATTAGAGATACGGCTGAAATAGGATTTTAGGGAG
AC_LY5 : TCCGCCCTTATCTTAC------AAGGAAAGGGAAAAAATGACATTATAT-----ATATAACATATTTAACATAGTTAAAGATAATTATAAACAAACCAAATCCTATTTATTTATTCTAATTAGAGATACGGCTGAAATAGGATTTTAGGGAG
AC_LY6 : TCCGCCCTTATCTTAC------AAGGAAAGGGAAAAAATGACATTATAT-----ATATAACATATTTAACATAGTTAAAGATAATTATAAACAAACCAAATCCTATTTATTTATTCTAATTAGAGATACGGCTGAAATAGGATTTTAGGGAG
AC_CF1 : TCCGCCCTTATCTTAC------AAGGAAAGGGAAAAAATGACATTATAT-----ATATAACATATTTAACATAGTTAAAGATAATTATAAACAAACCAAATCCTATTTATTTATTCTAATTAGAGATACGGCTGAAATAGGATTTTAGGGAG
AC_CF2 : TCCGCCCTTATCTTAC------AAGGAAAGGGAAAAAATGACATTATAT-----ATATAACATATTTAACATAGTTAAAGATAATTATAAACAAACCAAATCCTATTTATTTATTCTAATTAGAGATACAGCTGAAATAGGATTTTAGGGAG
AC_CF3 : TCCGCCCTTATCTTAC------AAGGAAAGGGAAAAAATGACATTATAT-----ATATAACATATTTAACATAGTTAAAGATAATTATAAACAAACCAAATCCTATTTATTTATTCTAATTAGAGATACGGCTGAAATAGGATTTTAGGGAG
AC_CF4 : TCCGCCCTTATCTTAC------AAGGAAAGGGAAAAAATGACATTATAT-----ATATAACATATTTAACATAGTTAAAGATAATTATAAACAAACCAAATCCTATTTATTTATTCTAATTAGAGATACGGCTGAAATAGGATTTTAGGGAG
AC_CF5 : TCCGCCCTTATCTTAC------AAGGAAAGGGAAAAAATGACATTATAT-----ATATAACATATTTAACATAGTTAAAGATAATTATAAACAAACCAAATCCTATTTATTTATTCTAATTAGAGATACGGCTGAAATAGGATTTTAGGGAG
AC_CF6 : TCCGCCCTTATCTTAC------AAGGAAAGGGAAAAAATGACATTATAT-----ATATAACATATTTAACATAGTTAAAGATAATTATAAACAAACCAAATCCTATTTATTTATTCTAATTAGAGATACGGCTGAAATAGGATTTTAGGGAG
AC_FS1 : TCCGCCCTTATCTTAC------AAGGAAAGGGAAAAAATGACATTATAT-----ATATAACATATTTAACATAGTTAAAGATAATTATAAACAAACCAAATCCTATTTATTTATTCTAATTAGAGATACGGCTGAAATAGGATTTTAGGGAG
AC_FS2 : TCCGCCCTTATCTTAC------AAGGAAAGGGAAAAAATGACATTATAT-----ATATAACATATTTAACATAGTTAAAGATAATTATAAACAAACCAAATCCTATTTATTTATTCTAATTAGAGATACGGCTGAAATAGGATTTTAGGGAG
AC_FS3 : TCCGCCCTTATCTTAC------AAGGAAAGGGAAAAAATGACATTATAT-----ATATAACATATTTAACATAGTTAAAGATAATTATAAACAAACCAAATCCTATTTATTTATTCTAATTAGAGATACAGCTGAAATAGGATTTTAGGGAG
AC_FS4 : TCCGCCCTTATCTTAC------AAGGAAAGGGAAAAAATGACATTATAT-----ATATAACATATTTAACATAGTTAAAGATAATTATAAACAAACCAAATCCTATTTATTTATTCTAATTAGAGATACGGCTGAAATAGGATTTTAGGGAG
AC_FS5 : TCCGCCCTTATCTTAC------AAGGAAAGGGAAAAAATGACATTATAT-----ATATAACATATTTAACATAGTTAAAGATAATTATAAACAAACCAAATCCTATTTATTTATTCTAATTAGAGATACAGCTGAAATAGGATTTTAGGGAG
AC_FS6 : TCCGCCCTTATCTTAC------AAGGAAAGGGAAAAAATGACATTATAT-----ATATAACATATTTAACATAGTTAAAGATAATTATAAACAAACCAAATCCTATTTATTTATTCTAATTAGAGATACGGCTGAAATAGGATTTTAGGGAG
